# Purifying selection constrains the evolution of *Juquitiba virus* in wild *Oligoryzomys nigripes* communities

**DOI:** 10.1371/journal.ppat.1013839

**Published:** 2026-01-20

**Authors:** Briana Spruill-Harrell, Alejandro Ponce-Flores, Evans Ifebuche Nnamani, Robert D. Owen, Michael A. Whitt, Colleen B. Jonsson

**Affiliations:** 1 Department of Microbiology, Immunology and Biochemistry, College of Medicine, University of Tennessee Health Science Center, Memphis, Tennessee, United States of America; 2 Centro para el Desarrollo de la Investigación Científica, Asuncion, Paraguay; 3 Núcleo de Biodiversidad, Facultad de Ciencias Exactas y Naturales, Universidad Nacional de Asunción, San Lorenzo, Paraguay; 4 Regional Biocontainment Laboratory, University of Tennessee Health Science Center, Memphis, Tennessee, United States of America; 5 Department of Pharmaceutical Sciences, College of Pharmacy, University of Tennessee Health Science Center, Memphis, Tennessee, United States of America; 6 Institute for the Study of Host-Pathogen Systems, University of Tennessee Health Science Center, Memphis, Tennessee, United States of America; Public Health Agency of Canada, CANADA

## Abstract

Juquitiba virus (JUQV) is endemic in *Oligoryzomys nigripes* across several South American countries and causes hantavirus pulmonary syndrome when transmitted to humans via infectious saliva or excreta. We developed a next-generation sequencing (NGS) pipeline to generate the first complete reference genome for assessing the genetic diversity of JUQV in *Oligoryzomys* populations inhabiting the Mbaracayú Biosphere Reserve within the Atlantic Forest of Paraguay. From 32 additional *Oligoryzomys* specimens, we obtained 17 S- and M-segment viral RNA (vRNA) genomes from lungs with 94–100% sequence coverage and 101 additional vRNAs with ≥80% genome coverage and ≥500x sequence depth from saliva, urine, lungs, heart, kidney, liver, and spleen. Phylogenetic and phylogeographic analyses showed that the Paraguayan JUQV is genetically distinct from the Brazilian JUQV lineage. Shannon entropy calculations of genetic diversity revealed that saliva and lung samples had higher entropy values than urine, kidney, spleen, and heart samples. The greater genetic diversity was driven in part by greater nucleotide, but not amino acid, diversity in persistently infected rodent samples compared to acutely infected ones. Genetic diversity varied across collection sites, although, given the continuous habitat matrix, there was no apparent reason for these differences. Fixed Effects Likelihood analysis of lung, saliva, and urine sequences suggested that purifying selection was the primary driver of evolution, with no evidence of positive selection. Only three of the 29 codons in the N protein and the glycoprotein (GP) were under purifying selection, and only Gn harbored nonsynonymous mutations. We tested two of the nonsynonymous mutations within the Gn for their effect on entry into Vero cells using VSV-pseudotyped JUQV GP; however, only V504I resulted in a significant reduction in entry compared to wild-type Gn. In summary, tissue source, field locale, and persistent infection were clear drivers of virus evolution.

## Author summary

The unpredictable nature of outbreaks of hantavirus pulmonary syndrome throughout the Americas has motivated field studies of the etiological agents of this disease (i.e., orthohantaviruses) in their reservoir hosts (rodents) to understand their maintenance within the host, potential spill-over into other wildlife or human populations, and the risk of outbreaks and pandemics. In contrast to other ecological biomes, such as the North American Southwest desert or Northern European forests, our group has been unable to find compelling evidence that climate, microhabitat, habitat disturbance, or predator removal affects the prevalence of orthohantaviruses endemic in sympatric rodents within the Atlantic Forest of South America. To gain further insight into the maintenance and shedding of orthohantaviruses, we developed and implemented a next-generation sequencing pipeline to characterize their genomes in wild rodent reservoir communities in the Atlantic Forest. We present the first in-depth examination of the intrinsic genetic diversity and evolution of the Juquitiba virus within its reservoir, *Oligoryzomys nigripes*. Our studies suggest that within-host interactions of virus and host are likely the most significant drivers of viral evolution.

## Introduction

Several rodent-borne viruses in the *Orthohantavirus* genus, *Hantaviridae* family, cause two clinically distinct diseases, hemorrhagic fever with renal syndrome (HFRS) and hantavirus pulmonary syndrome (HPS) [1–9]. Human cases of HFRS or HPS infection have only been reported for some of the rodent-borne viruses, and one shrew-borne orthohantavirus, *Bowe orthohantavirus* [10,11]. Transmission of orthohantaviruses from rodents to humans occurs through inhalation of aerosolized virus-contaminated rodent excreta [12]. Generally, each rodent reservoir species harbors a unique orthohantavirus species, and the geographical regions where HPS or HFRS cases occur are tightly associated with the ecology of the rodent species in the Americas and Eurasia, respectively [3,13–16]. For example, the Andes virus (ANDV), which causes HPS, is endemic in *Oligoryzomys longicaudatus* in the Southern Cone [17–20], while Bayou virus (BAYV), Black Creek Canal virus (BCCV), and Sin Nombre virus (SNV) are endemic in *Oryzomys palustris*, *Sigmodon hispidus,* and *Peromyscus maniculatus*, respectively, in North America. Herein, we focus on the Juquitiba virus (JUQV) that has been identified in *Oligoryzomys nigripes,* common to the Atlantic Forest regions of Uruguay [21], Brazil [22–24], eastern Paraguay and northeastern Argentina [25,26] and is associated with HPS in these regions [20].

*O. nigripes* is native to the Atlantic Forest, the second largest tropical rainforest in South America, which extends from the east coast of Brazil into eastern Paraguay and Misiones Province of Argentina. In the Mbaracayú Biosphere Reserve, located within the Atlantic Forest of eastern Paraguay, we have identified JUQV and Jabora virus (JABV) in sympatric *O. nigripes* and *Akodon montensis*, respectively, using a nested RT-PCR amplification strategy that employed blood or tissues [25]. In nature, the transmission of orthohantaviruses among rodents within a species is thought to mainly occur through aggressive behavior involving exposure to saliva and excreta, and cross-grooming, particularly among males, may be a primary route of infection [27–30]. Our field studies in Paraguay [25,31–33] concur with published findings that the prevalence in male rodents is much greater than in females [28–30,34]. Experimental studies of laboratory mice or rodent reservoirs confirm the importance of saliva and excreta in horizontal transmission [35–39]. For example, in longitudinal field studies of BAYV in *O. palustris* in Peach Point, Texas, the virus was shed in saliva and urine for at least three months [40]. Laboratory infections of *Sigmodon hispidus* with BCCV result in viral shedding in urine for 70 days after infection [38,41].

In the Mbaracayú Reserve, we experimentally tested two drivers that affect rodent community structure: food resources and the exclusion of terrestrial mammalian predators, to ask how these factors may affect the prevalence of JUQV and JABV within their rodent reservoirs, *O. nigripes* and *A. montensis*, respectively [42,43]. These experiments revealed that neither the addition of food resources nor predator exclusion affects the seroprevalence of JUQV or JABV in their reservoirs. Notably, these studies showed that different landscapes within a dense, biodiverse neotropical jungle had little effect on JUQV prevalence in O. nigripes, whereas A. montensis exhibited habitat-associated differences in JABV prevalence. However, these studies were limited by reliance on antibody screening using IFA and nested RT-PCR for virus detection. Hence, to further advance our understanding of the ecology and evolution of orthohantaviruses in wild rodent communities, we developed and optimized an amplicon-based next-generation sequencing (NGS) pipeline to elucidate the genetic diversity of orthohantaviruses within our longitudinal field studies. First, we optimized the NGS pipeline to obtain complete, full-length S-, M-, and L-segment genomic vRNA sequences (negative-sense strand packed in virions) for JUQV from one of the *O. nigripes* specimens collected in Mbaracayú, serving as the reference genome. We chose to focus on deep sequencing of the genomic vRNA to capture the apparent infectious virus population transmitted in saliva and urine, as well as systemically within the host. High-quality genomes from numerous specimens were obtained, with >80% sequence coverage and an average depth of coverage exceeding 500x in RNA from lungs, saliva, urine, heart, liver, kidney, and spleen. Herein, we present the genetic diversity of JUQV within each *O. nigripes* and across the population in the Mbaracayú and provide evidence for negative and neutral selection of this virus as the predominant driver of maintenance, spread, and thus evolution within its rodent host.

## Results

### Deep sequencing of JUQV S-, M-, and L-segments from *O. nigripes* TK184992

We developed an amplicon-based, NGS approach with specific primers and pools (S1 Fig, S1 and S2 Tables) based on our prior strategy [44] to sequence the negative-sense strand of S-, M-, and L-segment vRNAs for JUQV from the tissues of *Oligoryzomys* species collected in the Mbaracayú, a protected biosphere reserve within Paraguay. Our focus was on the vRNA as our goal was to characterize the vRNA genomes that are packaged into virions, transmitted systemically, and shed in urine or saliva by the rodent population. We iteratively tested and optimized the NGS pipeline using the lung sample from *Oligoryzomys nigripes* TK184992 and characterized the complete S-, M-, and L-segments of JUQV from this animal. The S-segment was 1,902 bp in length with an open reading frame encoding the nucleocapsid protein (N protein; 429 amino acids) at nucleotide positions 43–1,329 (GenBank accession no. OR184959). The M-segment was 3,675 bp with an open reading frame at nucleotide positions 52–3,468, encoding the GP precursor of 1,139 amino acids (GenBank accession no. OR184986). The L-segment was 6,564 bp in length with an open reading frame encoding the RNA-dependent RNA polymerase (2154 amino acids) at nucleotide positions 36–6,497 (GenBank accession no. OR184993). For all analyses reported, sequences obtained from TK184992 were used as the reference sequence except where noted.

### Detection of JUQV vRNA in tissues, urine, and saliva

**Fig 1:**
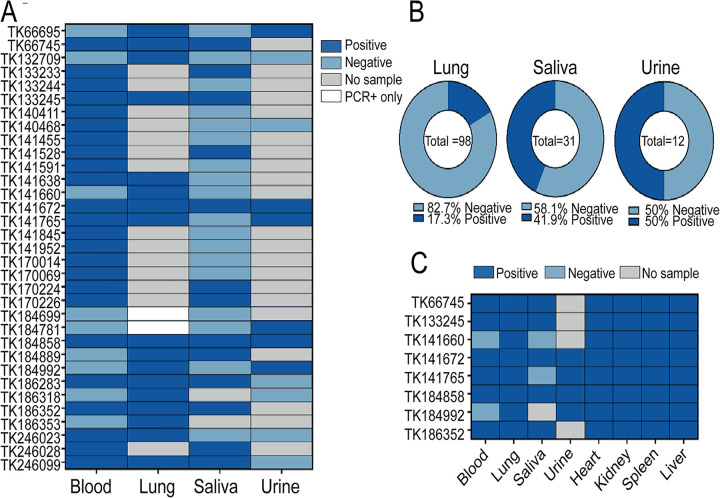
Summary of virus-positive tissues, saliva, and urine samples from *Oligoryzomys* spp. In **(A)**, we denote the samples identified to be positive (dark blue) or negative (light blue) for antibodies (whole blood) from prior work [33,42] and for viral RNA (lung, saliva, urine) herein using RNASeq and in **(B)** the overall percent positive or negative is presented. All samples are from *Oligoryzomys nigripes* except for one *O. mattogrossae*, TK66745. In **(C)**, we present data from the screening of mice, with the majority of sample types available, for those that were NGS-positive. The unique rodent TK identification is presented on the y-axis, and the sample type is on the x-axis in A and C. We set a cutoff of >1,000 reads mapped across a region of>500 bp of the S- and M-segment reference sequences to define a vRNA-positive sample. In light blue, we illustrate those samples that are negative. Grey indicates that no sample was available for screening. Two samples were previously reported as positive by PCR [32,41] and are highlighted in white.

The lungs from 98 *Oligoryzomys* rodents collected on six grids in the Mbaracayú over 18 kilometers were screened by NGS for the presence of JUQV S-, and M-segment vRNA using the reference TK184992 (S3 Table, S3 Fig). The grid maps and GIS coordinates for all rodents are included in our prior publications [33,42,43]. We set a cutoff of more than 1,000 reads mapped across a region of more than 500 bp of the S- and M-segment reference sequences to define a vRNA-positive sample. NGS screening revealed that 17 lung samples from adult males (inclusive of reference) were positive for JUQV vRNA, 16 from *O. nigripes,* and one sample from *O. mattogrossae* TK66745 (Fig 1). Of these 17 viral RNA-positive rodent specimens, seven were antibody-negative, suggesting an acute infection. The remaining ten vRNA-positive lung samples were previously reported as seropositive and were designated as persistently infected (Fig 1A). We previously reported that 23 *O. nigripes* and one *O. mattogrossae* were seropositive by IFA [33,42,43]. Two of these, TK184699 and TK184781, were previously reported as RNA^+^ but were not available for studies herein. Collectively from our 2014–2017 published studies of antibody prevalence and NGS screening studies herein for vRNA, a total of 33 *Oligoryzomys* specimens were identified as positive for antibody (blood) and/or viral RNA (lung) (Fig 1A). These included 32 adult males and one Ab^+^ adult female (TK141952 collected on Grid D). Of these, the one *O. mattogrossae* TK66745 was an adult male and was the only one of 54 (<2%) *O. mattogrossae* specimens that was seropositive [33,42,43]. In addition, we screened all available saliva (n = 31) and urine (n = 12) samples from these 33 individuals for JUQV S- and M-segment vRNA as these were the most easily detected by NGS as compared to L-segment. JUQV was present in 13 saliva (42%) and six urine (50%) samples (Fig 1B). This screening resulted in the detection of six additional samples that were vRNA and antibody positive, which were previously identified as only antibody positive and had no detectable lung vRNA [42,43].

We identified eight vRNA-positive individuals (seven *O. nigripes* and one *O. mattogrossae*) for which all tissues, saliva, and urine were available and screened by NGS for JUQV S- and M-segment vRNA. Reads from each organ were mapped to the respective lung consensus sequences from that rodent. The S- and M-segment vRNA sequences were detected in all organs tested, suggesting widespread infection of the heart, liver, kidney, and spleen (Fig 1C). Saliva (5/8) and urine (4/8) had fewer positives, but this could be due to limitations in the detection of our NGS pipeline for these sources.

### Phylogenetic characterization of JUQV from *Oligoryzomys* rodents in the Mbaracayú Reserve

**Fig 2:**
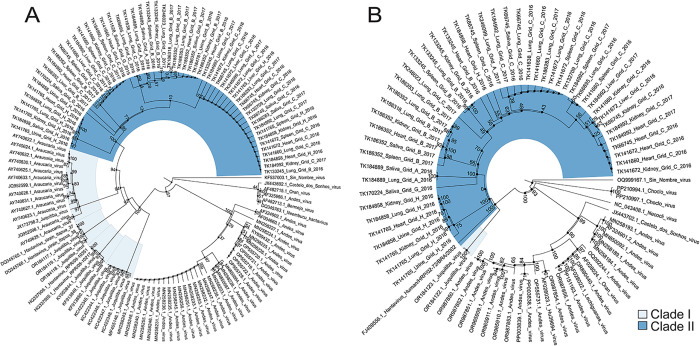
Phylogenetic relationships of JUQV S and M-segment coding regions and representative South American hantaviruses. **(A)** Maximum likelihood tree of the S-segment mRNA coding sequence (CDS; 1,065 bp after alignment and trimming corresponding to nucleotides 265-1329 of reference OR184959). *Orthohantavirus sinnombreense* (Sin Nombre virus; KF537003.1) was used as the outgroup, and phylogeny was inferred using the GTR + F + G4 model. **(B)** Maximum likelihood tree of the M-segment mRNA coding sequence (CDS; 3,420 bp after alignment and trimming corresponding to nucleotides 52-3468 of reference OR184986). *Orthohantavirus sinnombreense* (Sin Nombre virus; OQ999167.1) was used as the outgroup, and phylogeny was inferred using the TIM2 + F + I + G4 model. For visualization, branch lengths were proportionally rescaled in FigTree [46]; scale bar is not shown. Ultrafast bootstrap support values are shown at nodes. Sequences from this study are labeled by TK number, grid, year, and sample type. Two JUQV subclades are indicated in light blue (Clade I) and dark blue (Clade II). GenBank accession numbers and virus names are provided in the tree and the S4 Table.

To examine the genetic relationships among JUQV strains in this study, we constructed maximum likelihood phylogenetic trees using the coding regions of S- and M-segment nucleotide sequences with ≥80% genome coverage, derived from multiple tissues, saliva, and urine collected between 2014 and 2017 (S4 Table). All sequences from the Mbaracayú clustered within the JUQV clade (Fig 2). Phylogenetic analysis of Paraguayan JUQV sequences consistently resolved two well-supported subclades (bootstrap support ≥99%) in both S- and M-segment trees despite high overall similarity (Fig 2). Pairwise nucleotide identity across all S-segment sequences averaged 99.4% (range: 97–100%), with corresponding amino acid identity averaging 99.9% (range: 99.4–100%). Subclade structure for the S-Segment sequences from the Mbaracayú showed some association with collection site (Grid) but not year of collection or tissue source. Mbaracayú sequences formed a strong subclade (bootstrap 100%) that was distinct from sequences reported for Araucaria virus (Brazil), Itapúa virus (Paraguay, [45]), and other JUQV (Brazil). For the M-segment, the mean pairwise nucleotide identity was 99.0% (range: 96.4–100%), while amino acid identity averaged 99.9% (range: 99.5–100%). In the M-segment tree, JUQV sequences from Mbaracayú formed a distinct subclade separate from sequences from Itapúa (OR184122.1 and OR184123.1, [45]). Complete M-segment sequences were not available for other JUQV from Brazil. The within-host sequence characteristics are examined in a later section. Genomes recovered from *O. mattogrossae* (TK66745), which was captured on Grid C, formed a subgroup with sequences from *O. nigripes* also captured on Grid C, suggesting a likely spillover event from *O. nigripes* in that region.

The ARAV and JUQV sequences from Brazil also formed distinct subclades with the sequences from JUQV showing a closer relationship with ARAV (Fig 2). Since ARAV and JUQV sequences from Brazil were reported from collections 1000 miles from Paraguay, we decided to assess patristic distance of all the JUQV and ARAV sequences (S3 Fig). A graph of genetic and geographic distances from the patristic analysis suggested that all sequences were highly correlated (*r* = 0.923, *p* < 1 × 10 ⁻ ⁵). Of note, the ARAV and Paraguayan JUQV sequences showed less genetic distance than the Paraguayan JUQV versus the Brazilian JUQV sequences (distance = 0.2). These data suggest that 20% of the nucleotide sites vary between Paraguayan JUQV versus the Brazilian JUQV sequences, which is not apparent from pairwise identity comparisons.

### Shannon entropy reveals distinct patterns of genetic diversity across tissues, urine, and saliva

**Fig 3:**
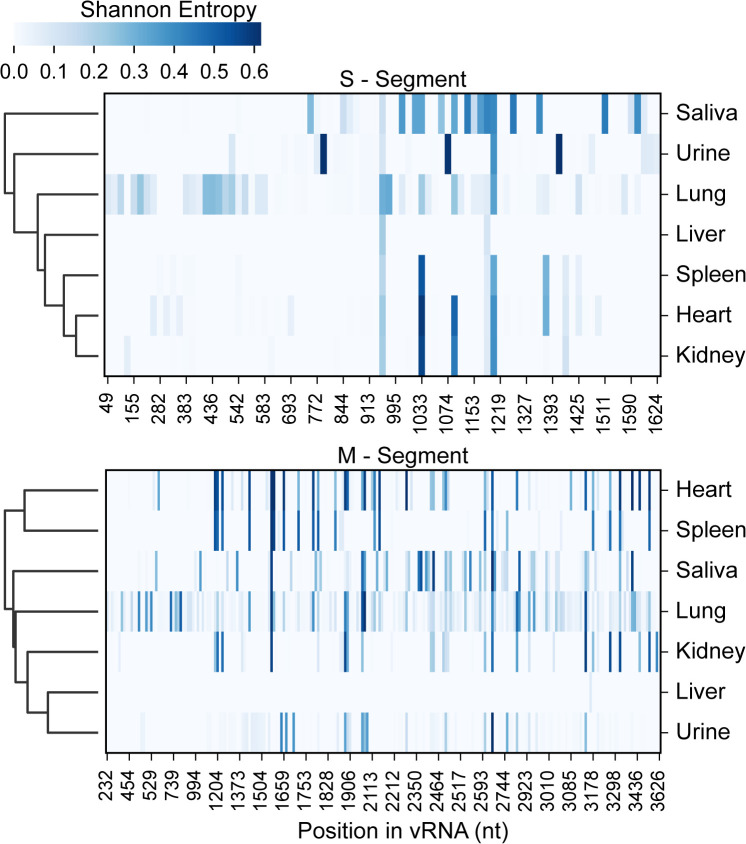
Heatmap of Shannon entropy for JUQV S- and M-segments and clustered by Shannon score for nucleotide. Entropy values reflect nucleotide diversity at each genomic position, with white indicating low diversity (conserved sites) and blue indicating high diversity (mutational hotspots). Hierarchical clustering of samples based on entropy profiles highlights similarities in genetic diversity. We used 19 S-segment NGS datasets in the analysis: TK132709, TK133245, TK141528, TK141638, TK141660, TK141672, TK141765, TK170224, TK184781, TK184858, TK184889, TK184992, TK186283, TK186318, TK186352, TK186353, TK246023, TK246099, TK66745. The M-segment included the same TK specimens, except for TK184781 and TK141528, which were not included because they did not meet the criteria (≥80% genome coverage with coverage depths ≥ 500x).

To explore the genetic diversity across different tissues, saliva, and urine, we calculated and clustered the Shannon entropy values from NGS data for each nucleotide in S- and M-segments from tissue, urine, saliva for all samples listed in Fig 1, and visualized using heatmaps with hierarchical clustering (Fig 3). Seventeen lungs (S5 Table), 13 saliva (S6 Table), six urine samples (S6 Table), and the eight kidneys, liver, spleen, and heart (S7 Table) were used in this analysis. However, before assessing genetic diversity using Shannon entropy, we defined an apparent variant frequency threshold for the NGS datasets by exploring SNPs at 1%, 2%, and 5% frequency thresholds (S2 Fig, S5–S7 Tables). Based on this assessment, we selected a 5% cutoff as a conservative threshold that balances sensitivity and specificity, while minimizing the inclusion of potential sequencing errors (i.e., a minimal number of SNPs are excluded).

The results revealed that S-and M-segment saliva and lung samples exhibited the highest entropy values. These sample types are clustered together in the heatmap, suggesting shared patterns of genetic variation, particularly for the S-segment (Fig 3). In contrast, kidney, spleen, and liver samples showed consistently lower entropy values, reflecting reduced genetic diversity. Cluster analyses suggested conserved hotspots of variability across all sample types. The clustering analysis further underscores the distinct diversity profiles of the sample types with greater genetic variability (saliva, lung) than those of the relatively conserved ones (kidney, spleen, heart, liver). The heart and spleen clustered together.

### JUQV polymorphisms in S- and M-segments from lungs, saliva, and urine

We further examined genomic diversity by focusing on SNPs present at ≥5% frequency in high-quality genomes (≥80% genome coverage with coverage depths ≥ 500x) from lung, saliva, and urine samples, using TK184992 as the reference (S6 Table). Polymorphisms were classified as consensus polymorphisms (≥50% frequency) or minority polymorphisms (<50% frequency).

**Fig 4:**
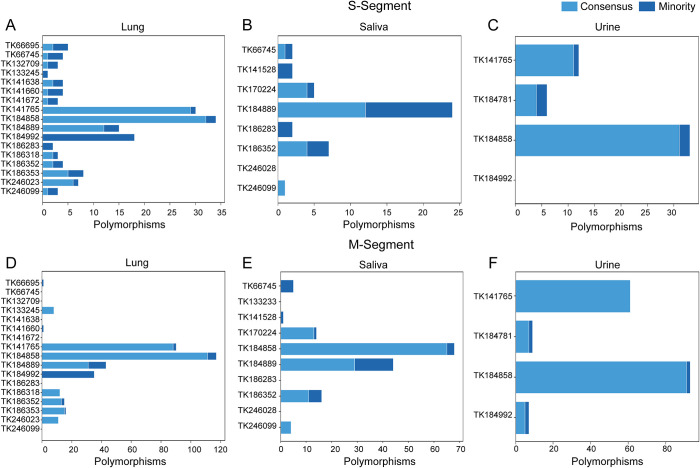
Number of consensus and minority polymorphisms in vRNA genomes from lung, saliva, and urine samples. Consensus polymorphisms (≥50% frequency, x-axis) are shown in light blue, and minority polymorphisms (<50% frequency) in dark blue. The y-axis provides the TK number for the sample type. TK141528, TK184781, and TK170224 data sets were not included as they did not meet the inclusion criteria (≥80% genome coverage with coverage depths ≥ 500x).

Lung samples harbored the highest number of unique S-segment (n = 53; Fig 4A) and M-segment (n = 158; Fig 4B) SNPs, compared to saliva (S = 30; M = 114; Fig 4B, E) and urine (S = 36; M = 105; Fig 4C, F). Notably, two lung samples, TK141765 and TK184858, had the highest number of polymorphisms, with the majority being shared; TK141765 (S=29/30, M = 90/90) and TK184858 (S=29/34, M = 90/117), suggesting that these variants may have been derived from a common ancestral sequence. These two samples were collected at the same site (Grid H) during February 2016. Similarly, M-segment genomes from individuals collected at Grid B (TK186318, TK186352, and TK186353) during the February 2017 sampling year shared many polymorphisms, suggesting close contact and potential virus transmission within this group.

While the M-segment consistently exhibited greater diversity than the S-segment across all tissues, compartment-specific trends were observed. Urine samples showed the highest median number of S-segment SNPs per genome (median = 9; range: 0–33), exceeding those in lung (median = 4; range: 1–34) and saliva (median = 2; range: 0–24). Similarly, urine samples also had the highest median number of M-segment SNPs (median = 35; range: 7–93), followed by lung (median = 8; range: 0–117) and saliva (median = 4.5; range: 0–68). Despite fewer overall SNPs, high per-sample diversity in urine may reflect localized replication or transmission bottlenecks.

Across all tissues and segments, most SNPs occurred at consensus frequencies, suggesting broad fixation within individual hosts (Fig 4A–F). A smaller proportion appeared only in the minority population or were detected as both consensus and minority variants across samples. Notably, saliva exhibited the greatest proportion of unique minority variants (S: 43%; M: 20%), compared to lung (S: 15%; M: 11%) and urine (S: 11%; M: 5%), indicating greater sub-consensus variability in this compartment. Saliva samples also harbored a higher proportion of private mutations (S: 73%; M: 81%), compared to lung (S: 19%; M: 27%) and urine (S: 58%; M: 41%), further supporting compartment-specific evolution. The number of consensus and minority polymorphisms in vRNA genomes from tissues and excreta reported by individual *Oligoryzomys* rodents is provided in S3 Fig

### Mutational burden

**Fig 5:**
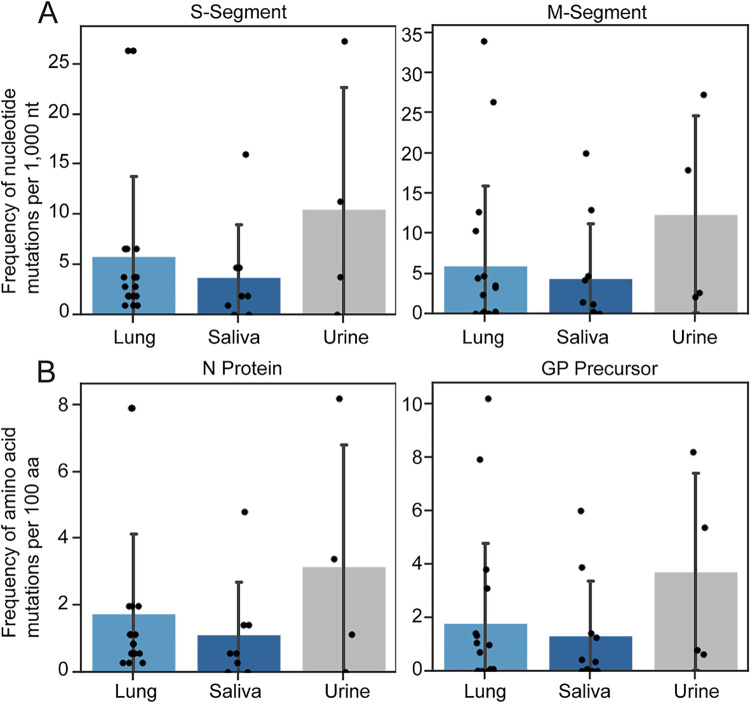
Frequency of JUQV S- and M-segment mutations. The frequencies of **(A)** SNPs per 1000 nt and **(B)** amino acid mutations per 100 amino acids (aa) are illustrated for lung, saliva, and urine sequences. The height of the bar represents the mean of the data set, and the error bars represent the standard deviation.

We estimated and compared S- and M-segment vRNA mutation frequency per 1,000 nucleotides (nt) or per 100 amino acids (aa) using sequence data from lungs, saliva, and urine by counting the number of coding region polymorphisms (S-segment cRNA regions 265–1329 and M-segment cRNA regions 52–3468) as compared to the reference genome (Fig 5). S- and M-segment genomes from the urine exhibited the highest average frequency of SNPs compared to genomes from the lungs and saliva (Fig 5A, B). We used the Kruskal–Wallis test to compare SNP and amino acid mutation frequencies across different sample types (lung, saliva, and urine) [47]. There was no significant effect of sample type on the frequency of S-segment SNPs or N protein mutations (H(2)=1.59, P = 0.45). Likewise, SNP frequencies in the M-segment did not differ by sample type at either the nucleotide level or the amino-acid level (H(2)=2.23, P = 0.33). Because none of the global tests reached significance, pairwise post-hoc comparisons using Dunn’s test [48] were not pursued. Taken together with the Shannon entropy analysis, these results indicate that while saliva and urine display broader positional diversity, the absolute burden of mutations per kilobase or per 100 amino acids does not differ appreciably among these sample types when a 5% variant-frequency threshold is applied. The apparent mutational frequency of the S- and M-segments ranges from 0.1-25 x10^-3^.

### Distribution of nonsynonymous mutations in orthohantaviral N protein and glycoprotein coding regions from lungs, saliva, and urine

**Fig 6:**
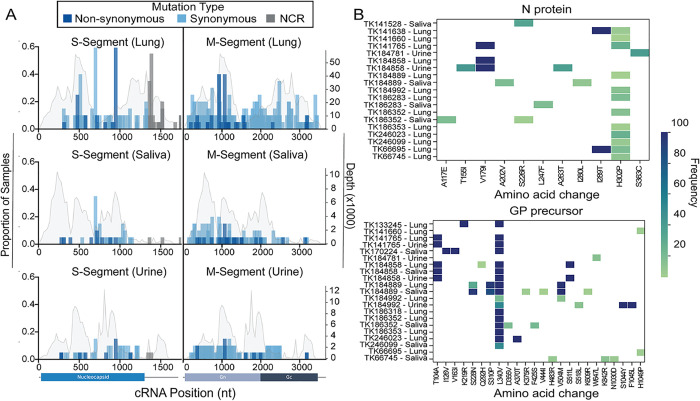
Distributions of SNPs in NCR and nonsynonymous mutations in coding regions of S- and M-segments. **(A)** Proportion of lung, saliva, and urine samples that share specific SNPs in the cRNA of S- and M-segments. The x-axis shows the nucleotide (nt) within the cRNA position, and the y-axis indicates the proportion of samples with a specific mutation. Each SNP is represented by a colored bar: synonymous (light blue), non-synonymous mutation (dark blue), or noncoding regions (NCR, grey). A grey line in the background of each plot represents the depth of coverage, with values indicated on the right y-axis. **(B)** Heat maps of nonsynonymous mutations in the JUQV N and GP display within-sample frequency of amino-acid substitutions across lung, saliva, and urine samples. Each row represents an individual genome and each column an amino-acid position; color intensity increases with mutation frequency (scale 0 – 100%).

We examined nonsynonymous mutations in the N protein and glycoprotein (GP) from lung, saliva, and urine samples. There was a total of 11 nonsynonymous amino acid changes in the N protein spanning nucleotide positions 392–1131 across 17 individual TK specimens (Fig 6A). Most N protein mutations occurred at minority variant frequencies (<50%) and were detected in fewer than two samples. Notably, H302P was detected at low frequency in most genomes, whereas V179I and I289T reached high frequency (>80%) in two individuals from Grid H (TK141765 [lung]; TK184858 [lung and urine]) and two individuals from Grid C (TK141638 [lung]; TK66695 [lung]), respectively, suggesting potentially emerging variants (Fig 6B).

There was a total of 24 nonsynonymous GP mutations spanning nucleotide positions 361–3197 across 17 individual TK specimens (Fig 6B). The majority of these mutations (79%) were in the Gn portion of the protein (1–652 aa), which is involved in cellular attachment and entry. L340V was consistently observed across tissue/saliva/urine, appearing at >50% frequencies (Fig 6B). Aside from T104A, which appeared in two different *Oligoryzomys* rodent samples from Grid H (TK141675 [lung, urine]; TK184858 [lung, saliva, urine]) and H1049P, which appeared in two individuals from Grid C (TK141660 [lung]; TK66695 [lung]), all other mutations were rare, suggesting limited transmission potential.

### Within-host genetic and amino acid diversity of JUQV

We used PopART (Population Analysis with Reticulate Trees) [49] to examine the within-host consensus sequence relationships for high-quality S- and M genomes for the NGS datasets from lung, kidney, heart, spleen, and saliva (and/or urine) of seven *O. nigripes* rodents (TK133245, TK141660, TK141672, TK184858, TK184992, TK186352) and one *O. mattogrossae* (TK66745; S7 Table). We focused on SNPs present at a frequency of≥5% in high-quality data with coverage depths of≥500 × .

**Fig 7:**
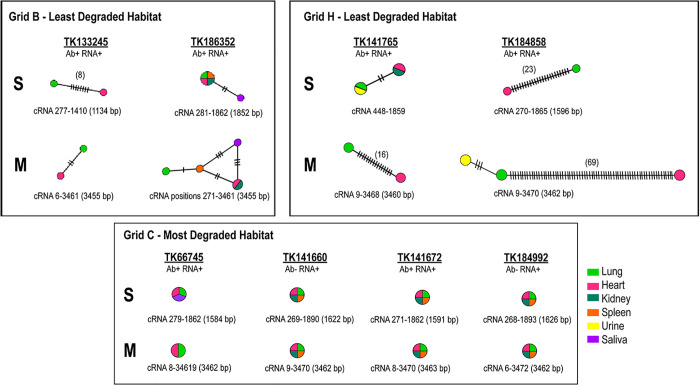
Within-host network analysis of JUQV S- and M-segment sequences from eight rodent specimens. For those samples with sufficient sequence integrity (≥80% genome coverage and ≥500x depth of coverage), we used the program PopArt to map within-host relationships. The samples chosen were from the set of eight specimens in Table 1C, which had the most significant number of available sequences of all those listed in Table 1A. Analyses were restricted to samples with long, contiguous nucleotide regions to maximize the number of shared informative sites for haplotype network reconstruction. The hash marks in the networks denote nucleotide differences between samples. Three areas (grids) were associated with these eight samples. As the samples were collected in areas with different levels of degradation this information was included from our prior published efforts [42]. The nucleotide regions analyzed are shown below each network and correspond to cRNA consensus sequences.

For most individuals, there were few nucleotide changes in the S- and M-segment consensus sequences across within-host sampled tissues (Fig 7). Five rodents (TK66745, TK141660, TK184992, TK141672, TK186352) showed nearly identical consensus sequences (<3 SNPs difference) in lung, heart, spleen, kidney, and/or saliva (Fig 7). However, despite this similarity at the consensus level, the minority variant populations in these individuals contained a higher number of SNPs than consensus variants present at ≥50% frequency, underscoring low levels of within-host diversity (S2 Fig). Minority variants were abundant across lung, spleen, heart, and kidney samples; however, nonsynonymous minority variants were particularly enriched in lung, saliva, and urine.

**Table 1:** Codon sites under purifying selection detected by Fixed Effects Likelihood across JUQV consensus sequences.

Protein	Codon	dS	dN	dN/dS	LRT	P value	Samples
**N protein**	157	17.4	0.0	3.8	2.8	0.02	Lung, Saliva/Urine
	158	189.9	0.0	3.2	3.715	0.03	Lung, Saliva/Urine
	206	12.3	0.0	3.7	2.283	0.05	Saliva/Urine
	237	40.1	0.0	3.6	3.57	0.04	Lung
	261	50.9	0.0	5.0	4.876	0.02	Lung, Saliva/Urine
	277	166.3	0.0	6.9	4.358	0.03	Saliva/Urine
	288	43.6	0.0	3.7	4.021	0.04	Lung
	316	14.7	0.0	2.9	2.834	0.04	Lung, Saliva/Urine
	322	15.9	0.0	3.8	2.787	0.05	Lung
	361	135.3	0.0	3.0	3.552	0.01	Lung, Saliva/Urine
**GP precursor**	50	23.6	0.0	2.9	4.692	0.03	Lung, Saliva/Urine
	62	40.1	0.0	5.7	8.219	0.00	Lung, Saliva/Urine
	84	15.3	0.0	2.8	4.047	0.04	Lung, Saliva/Urine
	119	35.6	0.0	2.7	4.124	0.03	Saliva/Urine
	149	45.5	0.0	3.3	4.077	0.03	Saliva/Urine
	159	11.9	0.0	4.0	4.259	0.04	Lung
	163	26.2	0.0	3.3	4.689	0.03	Lung
	180	37.5	0.0	3.2	3.853	0.03	Saliva/Urine
	205	268.5	0.2	8.9	5.749	0.04	Saliva/Urine
	232	13.9	0.0	3.9	4.754	0.03	Lung, Saliva/Urine
	292	175.5	0.0	3.7	3.882	0.05	Lung, Saliva/Urine
	299	16.3	0.0	4.1	5.056	0.02	Lung, Saliva/Urine
	380	20.8	0.0	3.7	3.876	0.03	Saliva/Urine
	381	23.6	0.0	3.2	4.508	0.03	Lung, Saliva/Urine
	480	53.8	0.0	4.2	8.3	0.00	Lung
	495	24.9	0.0	3.0	4.042	0.04	Lung, Saliva/Urine
	504	75.2	3.9	20.6	5.719	0.02	Lung
	618	30.4	0.0	6.7	3.914	0.04	Saliva/Urine
	619	8.6	0.0	2.3	2.517	0.05	Saliva/Urine
	654	30.5	0.0	3.6	5.938	0.01	Lung
	714	17.0	0.0	3.0	2.947	0.05	Saliva/Urine
	732	87.9	0.0	3.1	5.953	0.01	Saliva/Urine
	776	17.0	0.0	3.0	2.947	0.05	Saliva/Urine
	810	35.2	0.0	4.3	5.035	0.02	Lung, Saliva/Urine
	888	16.3	0.0	3.7	5.483	0.02	Lung
	891	18.7	0.0	4.4	5.18	0.02	Lung
	914	17.7	0.0	4.7	4.395	0.04	Lung
	962	61.5	0.0	7.6	8.951	0.00	Lung
	1118	33.4	0.0	6.4	8.378	0.00	Lung

dS = synonymous substitution rate. dN = nonsynonymous substitution rate. dN/dS = likelihood score for the null model. LRT = likelihood ratio test statistic.

One exception to the five rodents described above was TK184992, which harbored six consensus mutations in the M-segment sequences from urine, three of which were nonsynonymous (L340V [53% frequency], S1044Y [99% frequency], F1045L [99% frequency]). L340V was detected in both urine and lung sequences from this individual and was also found across the broader rodent population, suggesting it may represent a stable, circulating variant. Interestingly, urine from TK184992 contained two unique M-segment mutations (S1044Y and F1045L) that were not detected in the kidney sample, despite the urine having been filtered through this organ. This suggests that viral populations present in urine may differ from those detected in kidney tissue, potentially reflecting differences in viral replication or detection sensitivity between sample types.

Two animals captured at the same site (Grid H) three days apart in 2016 (TK184858 and TK141765), and one from Grid B in 2016 (TK133245), exhibited greater nucleotide divergence in the S- and M-segment consensus sequences from heart, kidney, and/or spleen compared to lung. In all cases, synonymous mutations predominated, consistent with neutral accumulation during replication, while only a few nonsynonymous changes were detected.

### Nucleotide, but not amino acid, mutation frequency differs between rodents with acute and persistent infection status

**Fig 8:**
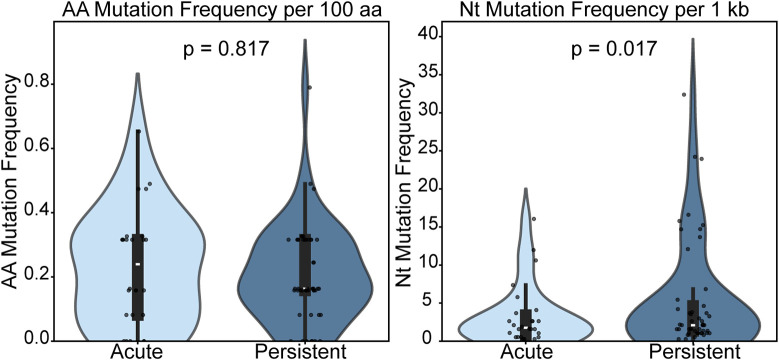
Mutation frequency distributions in acute and persistently-infected rodents. We compare the nucleotide (Nt) and amino acid (AA) mutation frequencies of orthohantaviral genomes in rodents with acute Ab-/RNA+) or persistent (Ab + /RNA+) infections. Statistical comparisons were performed using a one-sided permutation test (Persistent > Acute). Permutation tests were implemented in Python (version 3.10) using SciPy (version 1.11) with 10,000 random permutations.

To better understand the potential effect of antibody presence on genetic diversity, we analyzed the relationship between antibody presence and mutation frequency (Fig 8). Previous studies of wild rodent colonies report that it typically takes 10–14 days post-infection for IgG antibodies to become detectable [50]. Therefore, we classified rodents with robust antibody titers as “persistently infected,” reflecting an established infection. In contrast, those positive for viral RNA but without detectable antibodies were classified as “acutely infected” (i.e., within the initial 10–14 window post-infection before seroconversion). Next, we assessed the frequencies of nucleotide and amino acid mutations across lung, saliva, and urine samples. We initially checked for normality using the Shapiro-Wilk test and for variance equality using Levene’s test. The Shapiro-Wilk test indicated non-normal distributions in both acute and persistent groups for nucleotide and amino acid mutation frequencies (p-values < 0.001). In contrast, Levene’s test showed homogeneity of variance for amino acid mutations (p = 0.579) but unequal variance for nucleotide mutations (p = 0.035).

Given the skew introduced by many samples with zero mutations, we employed a one-sided permutation test (persistent (Ab-positive)> acute (Ab-negative)) to assess differences between the two groups. Permutation tests are well-suited to nonparametric distributions and can accommodate data skewness while remaining robust to heteroscedasticity. A comparison of genetic diversity frequencies between persistent and acute infections revealed a significant difference in nucleotide diversity (p = 0.0302), but not amino acid (p = 0.7075). These findings are visualized in Fig 8, which presents violin plots of the mutation frequency distributions. The plots show a clearly distinct distribution of nucleotide mutations between the two infection stages, whereas distributions of amino acid mutations largely overlap.

### JUQV evolution is driven by purifying selection

To assess whether positive or purifying (negative) selection is acting on JUQV consensus sequences, we applied the Fixed Effects Likelihood (FEL) method in Datamonkey. FEL tests the null hypothesis that the nonsynonymous substitution rate (*dN*) equals the synonymous substitution rate (*dS*) at each codon site. Rejection of this hypothesis indicates evidence of either purifying selection (*dN* < *dS*) or positive selection (*dN* > *dS*). Lung sequences (S:17; M:17) were analyzed as the primary dataset, while saliva (S:5; M:4) and urine (S:2; N:1) were analyzed separately as exploratory FEL screens due to limited sample size.

In the lung consensus genomes, FEL analysis indicated predominant purifying selection across both the partial N protein (encoded by the S-segment; aa 77–428) and the GP precursor (encoded by the M-segment; aa 1–1,133). Specifically, 8 of 352 codon sites (2.3%) in the N protein and 19 of 1,133 codon sites (1.7%) in the GP precursor showed significant evidence of purifying selection (p < 0.05, Table 1). No codon sites exhibited evidence of positive selection in either protein, or all other codons were under neutral selection. Analysis of saliva and urine sequences using FEL identified seven candidate codon sites in the N protein under purifying selection (aa 75–404; p < 0.05); five were also noted in lung sequences. In the GP precursor (aa 1–911), 19 codon sites were identified as significant (p < 0.05), with nine overlapping with those identified in lung (Table 1). Of those codons under purifying e selection, only three were associated with nonsynonymous changes: V163I, Q292H, and V504I.

### V504I mutation in JUQV Gn significantly reduces cellular entry

The orthohantavirus GP mediates entry into cells through receptor-mediated endocytosis [51]. We evaluated two nonsynonymous mutations that underwent significant purifying selection (p < 0.05): Q292H and V504I (Table 1). These amino acids are in regions of low conservation across Old and New World orthohantaviruses [52].

**Fig 9:**
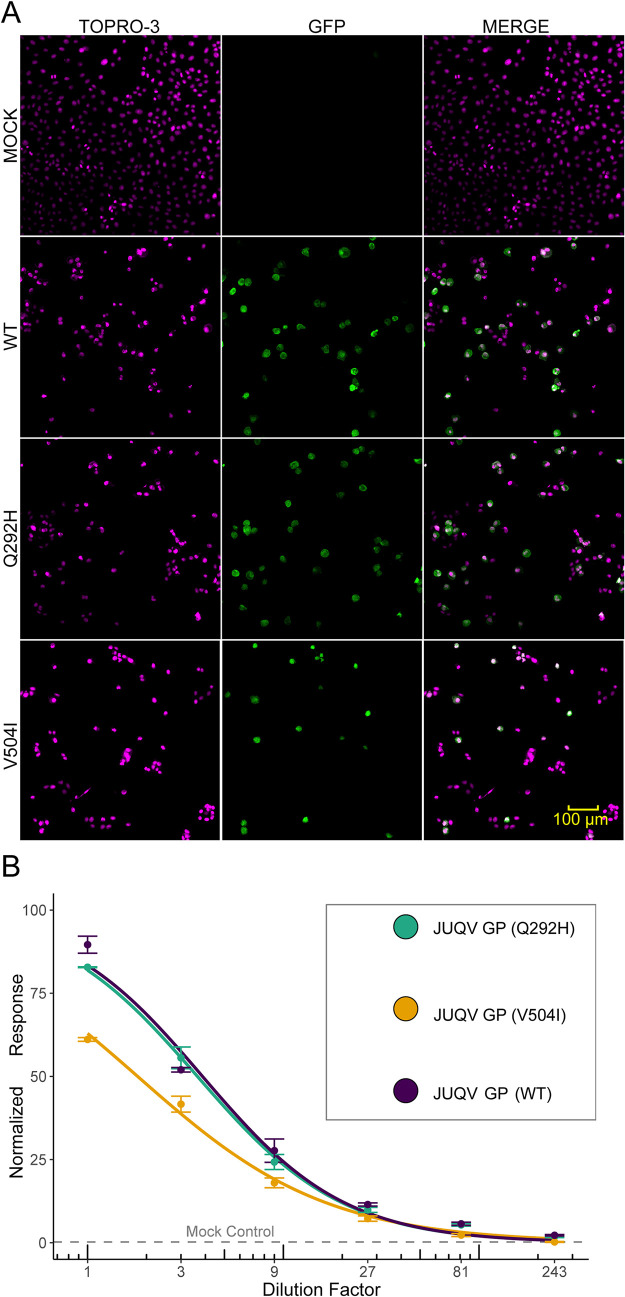
Assessment of JUQV GP entry using high content screening. **(A)** Representative confocal images are shown from the second dilution (3-fold) of JUQV WT GP, JUQV Q292H GP, and JUQV V504I GP, which were pseudotyped onto VSV-GFP. The three pseudotyped viruses were diluted to several concentrations to assess entry. Multiple images were automatically acquired for each well using the Yokogawa CQ1 at 20 × magnification. Shown are TO-PRO-3 nuclei (left), GFP signal (middle), and merged channels (right). Identical acquisition settings were applied across treatments. **(B)** The GFP signals were quantitated and used to construct dose–response curves for JUQV GP WT, Q292H, and V504I pseudotyped viruses. Data were normalized to the positive control (VSV at MOI 5) and background-subtracted. Data represent mean ± SEM of triplicates, fitted with a four-parameter logistic regression. Estimated EC₅₀ values of virus MOI (i.e., the effective MOI concentration for entry) were 3.17 (WT), 2.95 (Q292H), and 1.42 (V504I). The parameter estimates for (**B**) are given in S8 Table.

To assess the functional consequences of sites under purifying selection, we compared the entry efficiency of pseudotyped VSV particles carrying JUQV GPs with WT, Q292H, or V504I. We used high content confocal microscopy to measure entry of each as indicated by GFP expression from the pseudotyped VSVΔG-GFP vector (Fig 9A). GFP was quantified by cells and analyzed by four-parameter logistic regression (Fig 9B). The Gn Q292H mutant displayed a slightly lower EC₅₀ than WT (2.95 vs 3.17, p = 0.24), indicating no significant difference in entry efficiency. In contrast, the Gn V504I mutant exhibited an approximately two-fold reduction in EC₅₀ (1.42 vs 3.17, p < 2.2 × 10 ⁻ ¹⁶), consistent with a significant impairment of GP-mediated entry. Curve slopes were comparable across all conditions, supporting that the main effect was a shift in potency rather than changes in curve steepness.

### Structural modeling of Q292H and V504I mutations using AlphaFold

**Fig 10:**
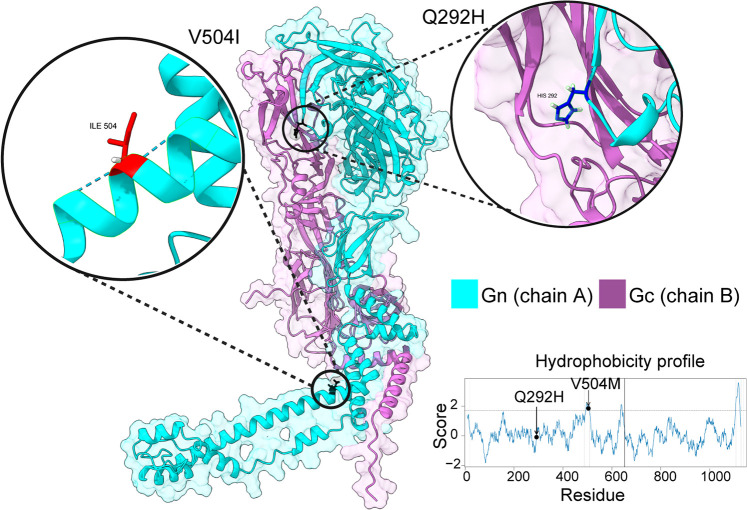
Structural comparison of JUQV and ANDV GPs using AlphaFold. The AlphaFold-predicted structure of the JUQV Gn (green) and Gc (purple) based on the ANDV GP prefusion complex (PDB ID: 6Y5F). The Q292H mutation is located near the fusion loop, a critical region for membrane fusion. In contrast, the V504I mutation is predicted to be within the transmembrane. This region was predicted with low confidence by AlphaFold and is absent in the ANDV crystal structure.

We employed AlphaFold2 modeling to visualize the Q292H and V504I mutations in the JUQV GP within the GP structure. Using the GP precursor sequence from TK184992 as a reference, we generated a JUQV GP structure using ColabFold v1.5.5: AlphaFold2 with MMseqs2 [53]. The resulting AlphaFold-generated JUQV structure was compared with the crystal structure of the ANDV GP prefusion complex, PDB 6Y5F [52] (Fig 10). Overall, the predicted JUQV structure aligned closely with the ANDV GP, validating the model’s accuracy for key regions. Notably, the Q292H mutation is located near the fusion loops, a critical area for mediating viral membrane fusion. In contrast, the V504I mutation resides within the predicted transmembrane region of Gn, which is a region with low structural confidence in the AlphaFold prediction and absent from the ANDV GP structure.

## Discussion

Surveillance of orthohantaviruses in wild rodent populations has been instrumental in revealing their global prevalence, for example, see [20,54–57]. The prevailing paradigm has been that *Orthohantavirus* species have coevolved [58,59] or cospeciated [60–62] with their rodent hosts over millions of years. A counterargument to the coevolutionary hypotheses is the repeated host shifts and speciation in closely related hosts, and these papers estimate the time to the most recent common ancestor (TMRCA) for the *Sigmodontinae-*borne hantaviruses to be 222–400 years before present [63,64]. However, few complete genome sequences exist for many of the strains circulating in South American rodents, which has the greatest diversity with more than 400 species of Sigmodontinae rodents in Central and South America [65]. The vast majority of studies have screened for antibodies to hantaviral antigens using ELISA or IFA to report prevalence in human populations or wildlife. Historically, the amplification of full-length genomes from wildlife samples for sequencing has been challenging due to the low amount of orthohantaviral RNA; hence, nested RT-PCR strategies are commonly used to obtain sequence information for phylogenetic analyses and genetic diversity [32,66,67]. These technical issues have resulted in hundreds of partial sequences for orthohantaviruses in GenBank which have been the main source of phylogenetic studies and hence evolutionary interpretation in the Americas. Of the orthohantaviruses listed in the online catalogues of the International Committee on Taxonomy of Viruses (ICTV) from the Americas in 2025, there are only eight with complete S-, M-, and L-segments (i.e., ANDV, BAYV, BCCV, Caño Delgadito virus, Choclo virus, Maporal orthohantavirus, Montano orthohantavirus, and SNV). Together with ANDV, the complete JUQV genome reported herein represents only two of the 15 unique genotypes in South America [20]; hence, other lineages and reassortments would add to the evolutionary perspective. This lack of sequence information creates a significant gap in our understanding of the evolution of orthohantaviruses, including their spillover into new species, such as humans, the mechanisms of host switching, how they maintain persistent infections in the reservoir host, their biology, and many other aspects. Recent advances in NGS of orthohantaviruses, along with the approach reported herein, will hopefully accelerate the search for answers to these questions [44,68,69].

A comparison of the similarity and phylogenetic assessments of the JUQV S- and M-segments from 17 lung samples of Oligoryzomys revealed high nucleotide (97–100%) and amino acid (100%) identities, except for TK66695 and TK141638, which had 99% amino acid identity. The S-segment sequences from Araucaria virus (ARAV) from Anápolis, Brazil, and Itapúa 37 and 38 from Itapúa, Paraguay, formed a subcluster with the JUQV sequences from Brazil, suggesting they represent a separate lineage from the JUQV circulating in Mbaracayú, as previously reported [70]. This group shows 88–96% nt and 98–100% aa identity. However, the M- and L-segments from the Brazilian JUQ viruses are not yet available, so the final phylogeny remains an open question. Moreover, the ARAV, and Itapúa 37 and 38 share distinct signatures not found in the Brazilian JUQV despite being separated by over 1000 miles, which includes crossing the River Paraná. As S- and L-segments reassort separately from M-segments [71,72], the evolutionary reconstruction of these lineages awaits the availability of M-segments. The JUQV sequences from Paraguay and Brazil have 80–82% nucleotide (nt) and 96% amino acid (aa) identity to ANDV, suggesting that these are distinct lineages. Previously, we reported the phylogenetic relationships of the S- and M-segments for all published complete coding sequences of hantaviruses in the Americas [25]. Based on this and data presented herein, these viruses share a common ancestor and are sister clades. Still, given the immense geographic distance between the areas they occupy, they are distinct evolving lineages (similar to *Orthohantavirus puumalaense*). Moreover, the incidence, clinical characteristics, and lethality rates of JUQV and ANDV differ in these geographical areas, with JUQV fatality in southern Brazil at 32.5% and ARAV in the Central Plateau and Southeast at 44.5% [16]. In comparison, HPS cases caused by ANDV in Argentina and Chile are associated with a lethality ranging from 21.4-35.9% [9]. Lastly, despite the broad distribution of *O. nigripes* in eastern Paraguay, only one HPS case has been reported that was attributed to JUQV [26]. Hence, despite the genetic, geographical, clinical, and ecological differences between ANDV and JUQV, these two viruses belong to the same lineage within the species *Orthohantavirus andesense*. A similar complex evolution and epidemiology occur with *Orthohantavirus puumalaense,* which has eight lineages [73], and *Orthohantavirus dobravaense,* which has four lineages [74]. As with these viruses, it will be essential to clarify and define lineage nomenclature for public health and research purposes.

Information on the systemic infection or shedding of orthohantaviruses in wild rodents [75,76] or in breeding colonies [50,77–79] of wild rodents is limited. Some of the most extensive studies have been conducted in an outdoor laboratory facility using wild-caught deer mice that were experimentally infected with SNV [50,77–80]. Combined, these studies detected viral antigen in the heart, lungs, kidneys, liver, spleen, brown adipose tissue, and salivary glands using IHC. At the same time, other organs had a low or negative antigen status [50,77–79]. In these studies, viral RNA and IgG (to the N protein) were detected in blood by 10–14 days post-infection, and in saliva between 15 and 90 days post-infection; however, no viral RNA was detected in urine or feces. In another study using these outdoor nesting boxes, deer mice were infected with SNV, and viral RNA/antigen were detected in the lungs, kidneys, and hearts as early as day 5 post-infection [50]. Viral RNA increased through day 20 in the lung and heart. In our studies, we identified wild *Oligoryzomys* that were positive for viral RNA, both with and without IgG antibody, by IFA. The majority of the virus-positive *Oligoryzomys* captured were adult males, and the one seropositive female adult had no detectable viral RNA. In the deer mouse studies mentioned above, sex and virus were not assessed. Viral RNA was detected in all tissues evaluated (lung, heart, liver, kidney), saliva, and urine. Our experience suggests that our success in detecting positives is related to field collection procedures and the duration of storage at -80 °C. We took great care to flash-freeze all samples in liquid nitrogen immediately upon collection. As our studies did not track individual mice but rather rodent communities, we are unable to provide a specific timing of infection. However, we can state that all our vRNA-positive samples consisted of adult males. As reported and reviewed by [81–84] and others, the transmission of orthohantaviruses by adult males is well established, likely due to virus transmission via urine and saliva during fighting.

In studies of *Orthohantavirus puumalaense* (PUUV) in its reservoir, the common bank vole [59,85,86], PUUV genetic diversity is highly correlated with ecology, geography, and landscape features. In our evaluation of genetic diversity, as determined by deep sequencing of the JUQV S- and M-segments across 17 *Oligoryzomys* rodents, the virus populations from lung, saliva, and urine were surprisingly similar at each locale, with no apparent difference by locale or habitat [42]. We hypothesize that this is due to the high biodiversity of the Atlantic Forest. However, as with other RNA viruses, the mutational burden of the viruses in the rodent community was high, ranging from 0.1 to 25 x 10^-3^ in S- and M-segments, and the number of nonsynonymous mutations averaged two amino acids for every 100. This mutational frequency is similar to that reported for SNV in deer mice, which ranged from 1.6 to 6.6 x 10^-3^ in a 389 nt section of G1 and 389 nt noncoding region of the S-segment [75]. Of note, in the *O. nigripes* minority SNP variants and amino acid diversity were significantly higher in saliva. Higher genetic diversity was also noted in the salivary gland in SNV [75]. Shannon-entropy profiling (Fig 3), which quantifies allele-frequency diversity independently of read depth, corroborated this observation: per-site entropy values were highest in saliva (and to a lesser extent urine), intermediate in lung, and lowest in the deeper visceral organs. Entropy heat-map clustering further showed that saliva and urine cluster together. At the same time, the lung forms an intermediate branch, and the kidney/spleen/heart/liver cluster separately, implying compartment-specific population structures that may arise from differing bottlenecks or selective pressures. While virus populations explore an enormous amount of sequence space across locales, the community’s consensus sequences reflect the substantial degree of purifying selection inferred by FEL for these viruses within the population and at the within-host level.

In their natural rodent reservoirs, orthohantaviruses establish long-term infections that favor persistence throughout the rodent’s life, although viral RNA is often difficult to detect [54]. The genetic diversity of JUQV varied significantly at the nucleotide level, with lower diversity observed during the acutely infected (vRNA, no IgG) stage compared to the IgG stage in individual rodents. However, synonymous mutations dominated during the IgG stage of infections, resulting in genetic variation without altering protein function. This dominance of synonymous changes likely reflects selective pressures for mutational robustness within the quasi-species, allowing JUQV to accumulate genetic diversity without compromising protein function [87]. While the effects of these changes on viral fitness remain to be studied, changes in viral RNA sequence may improve mRNA and/or vRNA stability, facilitate immune evasion, increase or decrease transmissibility, and thereby enhance the virus’s persistence and maintenance. This strategy has also been observed in other RNA viruses. For example, equine arteritis virus in carrier stallions undergoes extensive genome-wide purifying selection, with little evidence of adaptive amino acid changes during persistence [88]. Similarly, the foot-and-mouth disease virus in persistently infected cattle accumulates several non-synonymous mutations at specific capsid positions, but overall remains under purifying selection, showing no evidence of widespread antigenic escape [89]. In contrast, lymphocytic choriomeningitis virus persistence is driven by positively selected amino acid changes, such as the GP1 F260L mutation, which enhances receptor binding and facilitates the infection of dendritic cells; however, this change ultimately promotes chronic infection [90]. Fixed-effect likelihood analysis revealed that purifying selection acts uniformly on JUQV S- and M-segment consensus genomes. Two sites were identified to undergo purifying selection in the GP at a significance level of p < 0.05: Q292 and V504. We investigated the phenotypic consequences of the Q292H and V504I mutations on virus infectivity by generating VSV pseudotyped with the JUQV GP carrying either mutant. The JUQV GP:V504I pseudotyped virus had reduced cellular entry into Vero E6 as compared to the WT, whereas the JUQV GP with the Q292H was similar to the WT.

In conclusion, we report the first development and deployment of an NGS amplicon sequencing pipeline for the study of JUQV in wild *O. nigripes* rodents, an etiological agent of HPS [26] from rodent communities within the Mbaracayú Atlantic Forest within Paraguay, a tropical rain forest second in biodiversity to the Amazon [91]. In addition to *O. nigripes*, we also screened two other rodent species by NGS that we had previously detected antibodies to hantaviral antigens, *O. mattogrossae, and Hylaeamys megacephalus.* Of these, only one *O. mattogrossae* was positive for JUQV RNA. We have no evidence at this time that JUQV might persist in this species. An extensive survey of small rodents in northern New Mexico revealed a high prevalence of SNV across numerous mouse, woodrat, and gopher species tested [76]. Of the 11 native rodent species belonging to the family Cricetidae that have been identified in our studies, only O. nigripes and *O. mattogrossae*, *H. megacephalus*, and *A. montensis* were IFA positive [33,42,43]. Given the greater habitat diversity in the Atlantic Forest, this is probably not too surprising. In our studies of the habitats of the 11 rodent species in the Atlantic forest, we noted that most species were found at times in some of the same microhabitats [92]. The three most common species, accounting for 95.6% of captures and including *O. nigripes*, were encountered in several of the same microhabitats; however, this occurred significantly less frequently than expected by chance, and we concluded that *O. nigripes* is avoiding either the other two species or their preferred habitats. Using NGS datasets from organ, urine, and saliva samples collected from these *O. nigripes* rodent specimens, we present the phylogenetic relationships of JUQV in Paraguay and Brazil, which clearly underscore the need for further sampling along the Atlantic forest to elucidate the evolution of hantaviruses in South America. The vRNA diversity in wild rodent communities we noted, as well as within-host genetic diversity analysis, suggests that, despite high genetic diversity, amino acid diversity is highly constrained by purifying selection. Our study also suggests that persistent infections exhibit greater nucleotide diversity; however, most nucleotide-level changes were synonymous and are invisible to T & B cell responses, and organs would not be protected differently from these responses. Hence, there must be a different mechanism to explain the organ-specific entropy patterns. We acknowledge the limitations of our studies, which were confined to 17 S- and M-segments. However, with the success of this NGS platform, further sampling is expected to refine the results presented herein. In conclusion, much remains to be learned in studies of orthohantaviruses in wild rodent populations, such as the dynamics of JUQV systemic infection, its replication in salivary glands, how host factors promote infection (or are not present) or selection (winners and losers), and what mechanism drives the apparent diversity in lung and saliva, which warrants future investigation.

## Materials and methods

### Oligoryzomys samples

Tissue, urine, and saliva samples (S1 Table) were collected from August 2014 to February 2017 in Paraguay, as detailed in [33,42,43], including databases for each collected rodent with GIS coordinates and grid maps. In these field collections, tissues and fluids were harvested and immediately flash-frozen in liquid nitrogen before being transported to the United States, where the samples were stored at -80 °C. Species identification was confirmed by morphology and/or by molecular confirmation as described [33].

### Total RNA isolation from rodent tissues and fluids

Total RNA was isolated from tissue samples using the MagMAX mirVana total RNA isolation kit (Thermo Fisher). Approximately 10 mg of sample was cut from each tissue on dry ice. Frozen tissue was processed with 200 µL of lysis binding mix in bead mill tubes (Omni International) containing 2.8 mm ceramic beads (lung) or 1.4 mm ceramic beads (liver, kidney, spleen, and heart). Tissue was homogenized three times using the Bead Rupter 4 Homogenizer (Omni International) at 5 m/s for 10 s each. One hundred microliters of tissue homogenate were used for automated extraction on the KingFisher according to the manufacturer’s protocol (Thermo Fisher). RNA was eluted in 50 µL of elution buffer, heated to 65°C for 5 min, and stored at -80 °C until use. RNA was quantified on the Qubit® Fluorometer using the RNA BR Assay Kit (Thermo Fisher).

Total RNA was isolated from approximately 400 µL of saliva swabs or urine samples using the MagMax Viral Pathogen Kit (Thermo Fisher) on the KingFisher, following the manufacturer’s protocol. RNA was eluted in 50 µL of elution buffer, heated to 65°C for 5 min, and then immediately placed on ice or stored at -80 °C until use.

### cDNA synthesis

Five hundred nanograms of total RNA were used to generate a cDNA library using the Superscript IV First Strand Synthesis kit (Thermo Fisher) according to the manufacturer’s protocol with the optional RNase H step. For saliva and urine samples, 5 µL of total RNA was used to generate cDNA libraries. cDNA was synthesized in 20 µL reactions using multiplexed forward primer pools for vRNA-specific amplification (S2 and S3 Tables). Primers were added to the reaction to a final concentration of 0.03 µM per primer. cDNA products were stored at -80ºC until use.

### Library preparation and sequencing

PCR amplification was performed in five amplicon pools, with a total volume of 20 µL (S1 Fig), using 2.5 µL of cDNA and 12.5 µL of 2X Platinum SuperFi PCR Master Mix (Thermo Fisher). Forward and reverse primers (S2 and S3 Tables) were added to the reaction to a final concentration of 0.03 µM per primer. Initial denaturation was at 98°C for 30 s, followed by 30 cycles at 98 °C for 10 s, 60 °C for 30 s, 72°C for 15 s, and a final extension at 72 °C for 5 min. PCR products from each reaction were combined and then purified using a 0.8x ratio of AMPure XP beads (Beckman Coulter). DNA was quantified using a Qubit Fluorometer using the DNA High Sensitivity (HS) Assay Kit (Invitrogen). DNA was diluted to 1 ng/µL using UltraPure DNase/RNase-Free Distilled Water (Thermo Fisher) and 5 µL was used as input for library prep.

Libraries were prepared using the Nextera XT DNA Library Prep Kit (Illumina) according to the manufacturer’s protocol with the exception that libraries were double-size selected to obtain an average library size of approximately 450 bp. Right-sided size selection was performed using a 0.5x ratio and left-sided size selection was performed using a 0.7x ratio. The supernatant was discarded, the beads were washed, and the libraries were eluted in 15 µL of resuspension buffer.

The quality and average length of libraries were assessed with the 5200 Fragment Analyzer (Agilent Technologies) using the NGS fragment kit (Agilent Technologies). DNA was quantified on the Qubit Fluorometer using the DNA HS Assay Kit (Thermo Fisher). Libraries were diluted to 4 nM and pooled. Denatured and diluted libraries were combined with a 5% Spike-in control of PhiX (Illumina). Libraries were sequenced using the MiSeq Reagent Kit v3 (150 cycles) on a MiSeq sequencer (Illumina). For each sequence run, up to 24 samples were evaluated on a single cartridge. This was done to minimize cross-contamination and achieve sufficient read depth (approximately 1–2 million reads/sample).

### Genome assembly and low-frequency variant detection

Paired-end reads generated and sequenced on the Illumina MiSeq platform were processed using CLC Genomics Workbench v.21.0.3. Reads were trimmed to remove short reads (<50 bp) and low-quality bases (quality limit <0.05) and were mapped to S and M reference sequences obtained from TK184992 (GenBank accession nos. OR184959 (S), OR184986 (M), OR184993(L)). Reference-guided consensus sequences were generated from mapped reads with samtools v1.16.1 and bcftools v1.19 [93], applying thresholds of ≥500 × depth for variant calls, ≥ 50% allele frequency, and an ambiguity threshold of 0.5, with low-coverage regions (<10×) masked using bedtools2 v2.30.0 [94]. The consensus sequences of the 135 S- and M-segments of JUQV were deposited in GenBank (S4 Table).

Variants were identified using iVar v1.3.1 [95] following a comprehensive primer processing workflow that included initial primer trimming (iVar trim; minimum trimming quality of 50, base quality of 20, and sliding window size of 4), consensus genome generation, read realignment to the consensus sequence, secondary primer trimming on realigned reads. Primer mismatches were detected and problematic amplicon removal using iVar getmasked. Final variant calling was performed against the original reference genome using minimum thresholds of 1% frequency, ≥ 500x read depth, and base quality ≥30, with exploratory analyses conducted at 1%, 2%, and 5% frequency thresholds to assess sensitivity and specificity trade-offs. Following this evaluation, a 5% frequency threshold was adopted for all subsequent analyses. Bases were called above a 50% frequency in the consensus with an ambiguity threshold of 0.5.

### Phylogenetic and phylogeographic analysis

Nucleotide sequences of the S-segment (positions 265–1329 in reference OR184959) and M-segment (positions 52–3468 in reference OR184986) from this study, together with South American orthohantavirus sequences from GenBank, were aligned using MAFFT v7.526 with the --globalpair and --maxiterate 1000 parameters to generate high-quality multiple sequence alignments. Aligned sequences were filtered to retain only those with ≥80% coverage relative to the full alignment length. Phylogenetic trees were inferred using IQ-TREE 2.4.0 [96], with automatic model selection (-m TEST) [97] and ultrafast bootstrap (-bb 1000) [98]. Identical sequences were automatically identified and collapsed during likelihood computation and retained in the final tree to preserve taxon information. The best-fit model was selected based on BIC. The final tree was visualized and proportionally rescaled in FigTree v1.4.4 [46].

For phylogeographic distance analysis, we used APE (Analysis of Phylogenetics and Evolution) [99]. Pairwise genetic distances were calculated from the S-segment phylogeny (lung samples only), including JUQV sequences generated in this study, together with external JUQV sequences from Brazil and Paraguay (including Itapúa strains) and ARAVs. Geographic distances were computed from capture coordinates or the most precise available locality descriptions. Correlation between matrices was tested with a Mantel test (Pearson, 1e + 05 permutations).

### Mutation frequency analyses

The apparent mutation frequency per 1,000 nucleotides (nt) or 100 amino acids (aa) was estimated by counting the number of coding region SNPs (S-segment cRNA regions 250–1329 and M-segment cRNA regions 52–3461) relative to TK184992 reference sequences or the lung consensus sequence from each rodent. Genetic variations were compared using the non-parametric Kruskal-Wallis test [47]. Multiple pairwise comparisons were made using Dunn’s test [48].

### Nonsynonymous (dNS) and synonymous (dS) substitution rates

To identify amino acid sites under positive or purifying selection, MUSCLE alignments of complete coding regions from the mRNA sense of the S-segment (270–1329 nt) and M-segment (52–3459 nt) consensus lung sequences reported herein (n = 17) were aligned (GenBank accession no. OR184959-OR184992). Additionally, unique S- and M-segment consensus sequences from the kidney (n = 1), heart (n = 2) and saliva (n = 1) were included in the alignment (see S4 Table for GenBank numbers corresponding to: TK66745: Saliva_S, Saliva_M; TK141528: Saliva_S, TK184781: Urine_S; TK184858: Urine_S, Saliva_M, Urine_M; TK184889: Saliva_S, Saliva_M; TK186283: Saliva_S, TK186352: Saliva_S; TK170224: Saliva_M). Sequences were analyzed by the DataMonkey 2.0 web server [100] using fixed-effects likelihood (FEL). FEL infers nonsynonymous and synonymous substitution rates per site and is recommended for small sample sizes [101]. Identical sequences were removed, which left 19 S-segment sequences and 13 M-segment sequences for analysis.

### Shannon entropy calculations for genetic diversity

Shannon entropy was used to quantify the diversity of alleles at each genomic position in the S- and M-segments. For each position, allele frequencies for the reference and alternate alleles were calculated as the proportion of reads supporting each allele relative to the total read depth. Positions with missing data or zero depth were excluded from the analysis. The entropy for each position (*H*) was calculated using the formula:


$$H=\sum_{i}pi\;\times \text{ log}(p_{i})$$

Where $p_{i}$ represents the frequency of each allele (reference or alternate). Entropy values were calculated using the scipy.stats.entropy function [102] with the natural logarithm (${log}_{e}$) as the base. Cases where allele frequencies were zero were handled by setting their entropy contribution to 0. Entropy values reflect the degree of diversity at each site, ranging from 0 (no diversity, dominated by a single allele) to a theoretical maximum of log(*n*), where *n* is the total number of possible alleles.

### Network analysis

Reference-guided consensus sequences were generated from mapped reads as described above. For each individual rodent, the lung consensus sequence was used as the representative sequence. Multiple sequence alignments of consensus sequences from tissues, saliva, and urine of eight individuals (TK133245, TK141660, TK141672, TK184858, TK184992, TK186352, and TK66745) were constructed using MUSCLE with default parameters in MEGA X [103]. NEXUS files containing metadata were exported into PopArt v.1.7 [49] to infer a minimum spanning network using the algorithm described in [104] with default parameters, including an epsilon value of 0.

### Plasmids, cells, and generation of VSV pseudotyped JUQV

Plasmids (pCAGGS-MCS) expressing the wildtype (WT) JUQV glycoprotein (GP) from TK184992 (OR184986) or plasmids with mutant GPs, V504I, or Q292H were synthesized by GenScript. Transfection of cells with plasmids expressing JUQV GPs and infection with vesicular stomatitis virus (VSV) pseudotyping vector, VSV∆G-GFP, were performed as described previously [105,106], except that human embryonic kidney 293 cells containing SV40 T-antigen (HEK-293T, ATCC CRL3216, Manassas, VA, USA) were used instead of BHK cells, and FuGENE® 4K (Fugene, Madison, WI, USA) was used for transfections. HEK-293T cells were maintained with DM-10 containing Dulbecco’s modified Eagle medium (DMEM) plus Glutamax (Life Technologies, Grand Island, NY, USA) and 10% heat-inactivated fetal bovine serum (Hi FBS) (Life Technologies, USA). The VSV-G monoclonal I1 was used [107] for detection of VSV GP, and the cell line was maintained in Roswell Park Memorial Institute (RPMI 1640) (Life Technologies, USA) containing 10% Hi FBS.

### JUQV entry studies

Approximately 1.2 × 10⁴ Vero E6 cells were plated per well into 96-well plates (Greiner, Bio-One, Frickenhausen, Germany) in MEM containing 10% heat-inactivated FBS, 24 h before infection. Cells were washed with DPBS and infected with pseudotyped VSV carrying JUQV GPs or VSV-G stock for 1 h at 37 °C and 5% CO₂. The VSVΔG-GFP genome expresses GFP for microscopic detection in the cytoplasm. Negative controls included supernatants from mock-transfected cells. Each condition was assayed in triplicate using a serial dilution series (undiluted, 1:3, 1:9, 1:27, 1:81, 1:243). After 1 h, the inoculum was removed, and fresh MEM containing 10% FBS was added. Cells were incubated at 37 °C and 5% CO₂ for 24 h, washed, and fixed with 4% paraformaldehyde (Alfa Aesar, Ward Hill, MA, USA) for 15 min at room temperature. Fixative was quenched with 50 mM NH₄Cl for 15 min. Nuclei were counterstained with TO-PRO-3 (Invitrogen, Eugene, OR, USA). Plates were protected from light and stored at 4 °C until imaging on the Yokogawa CQ1 system.

### Immunofluorescence confocal microscopy and image analysis

High content confocal imaging was performed on the Yokogawa Confocal Quantitative Image Cytometer (CQ1) to detect GFP and TO-PRO nuclear signal. For each well, the CQ1 software (v1.07.01.01) was used to segment cells and nuclei and to export per-object measurements, including mean and median intensities for each channel, as well as morphological traits. Exported data were processed in R v4.4.2. Aggregates and debris were excluded based on nuclear circularity, area, and intensity. Infection status was defined by setting a threshold at the 99.9th percentile of GFP intensity from mock-infected controls. Counts of GFP-positive (infected) and GFP-negative (uninfected) cells were obtained per well (triplicates), and the ratio of infected cells was used to generate dose–response curves. A four-parameter logistic (4PL) regression was fitted to each dilution series to determine the 50% effective concentration (MOI) EC₅₀ for entry of JUQV WT GP and the mutant GP VSV pseudotyped viruses.

## Supporting information

S1 Fig Amplicon tiling strategy for JUQV genome next generation sequencing. (DOCX)

S2 Fig Distribution of unique single nucleotide polymorphisms in the S- and M-segment genomes. (DOCX)

S3 Fig (A) Patristic analysis of JUQV and ARAV nucleotide sequences and map (B) showing location of samples sequenced. (DOCX)

S4 Fig Number of consensus and minority polymorphisms in vRNA genomes from tissues and excreta of individual Oligoryzomys rodents. (DOCX)

S1 Table JUQV forward primers for the S-, M-, and L-segments are listed along with their associated primer pools. (DOCX)

S2 Table JUQV reverse primers for the S-, M-, and L-segments are listed along with their associated primer pools. (DOCX)

S3 Table *Oligoryzomys spp*. samples used in this study. (DOCX)

S4 Table JUQV Genbank accession numbers. (DOCX)

S5 Table Genome coverage and average depth of coverage of JUQV S- and M-segment vRNA from *Oligoryzomys* lungs. (DOCX)

S6 Table Genome coverage and average depth of coverage of JUQV S- and M-segment vRNA from *Oligoryzomys* saliva and urine. (DOCX)

S7 Table Genome coverage and average depth of coverage of JUQV S- and M-segment vRNA from *Oligoryzomys* heart, kidney, spleen, and liver. (DOCX)

S8 Table Parameter estimates (Estimate ± SE) for JUQV GP variants. (DOCX)

S9 Table Listing of the TK numbers of 33 individual Oligoryzomys mice listed in Fig 1* of this paper. (DOCX)
