# Supplementary material for: Purifying selection constrains the evolution of Juquitiba virus in wild Oligoryzomys nigripes communities
Source: PLoS Pathog. 2026 Jan 20;22(1):e1013839. doi: 10.1371/journal.ppat.1013839 (PMC12844527; doi:10.1371/journal.ppat.1013839)
Supplement: S1 Fig — (DOCX) [file ppat.1013839.s001.docx]

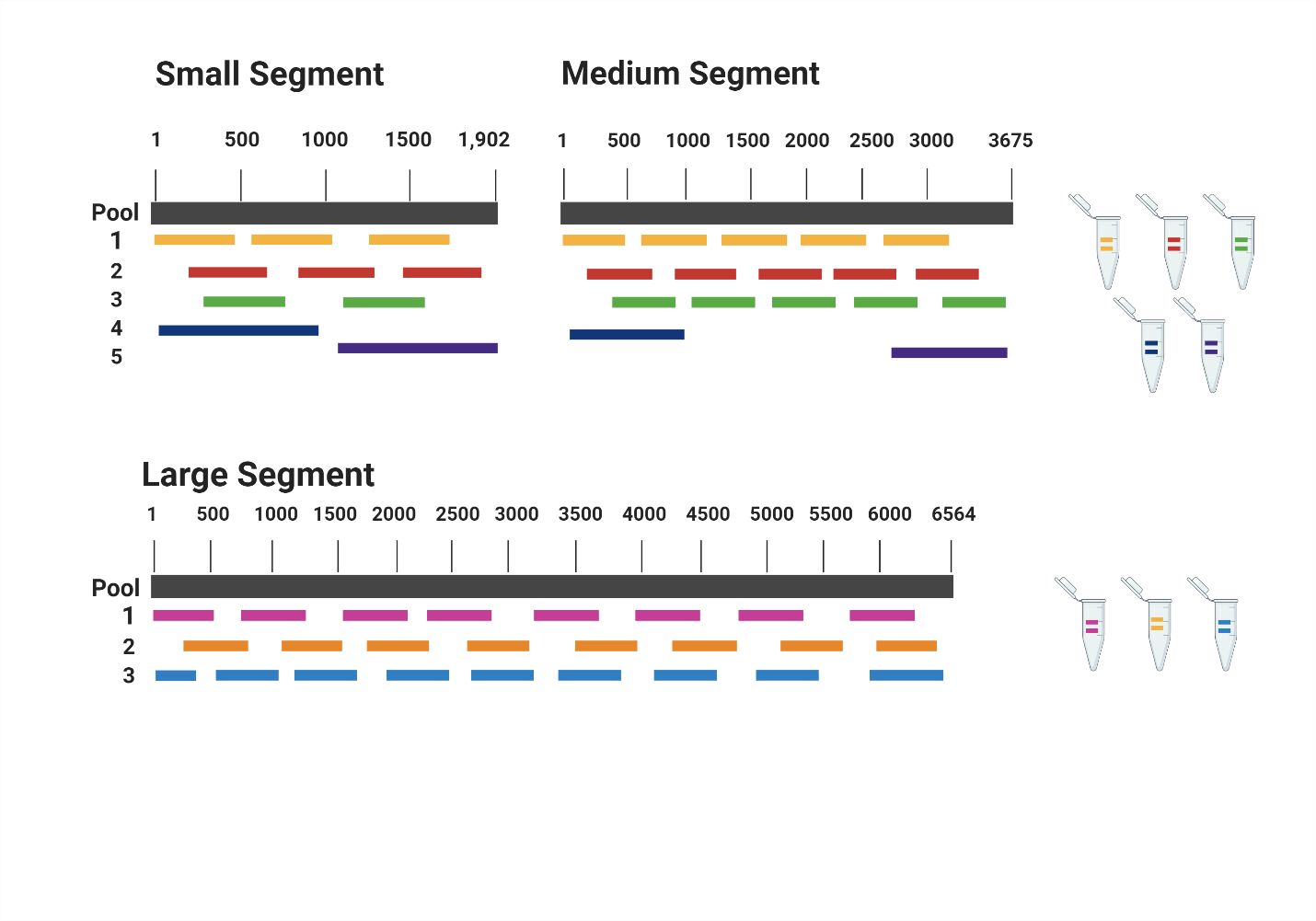


**S1 Figure. Amplicon tiling strategy for JUQV genome next-generation sequencing**. Illustration of the S, M, and L genome segments shows colored bars representing the amplicons generated by each primer set. Each color corresponds to a specific primer pool used for amplification.  Created in BioRender. Spruill-Harrell, B. (2026) [https://BioRender.com/4youkc1.](https://biorender.com/4youkc1)
