# Supplementary material for: Purifying selection constrains the evolution of Juquitiba virus in wild Oligoryzomys nigripes communities
Source: PLoS Pathog. 2026 Jan 20;22(1):e1013839. doi: 10.1371/journal.ppat.1013839 (PMC12844527; doi:10.1371/journal.ppat.1013839)
Supplement: S2 Fig — (DOCX) [file ppat.1013839.s002.docx]

**
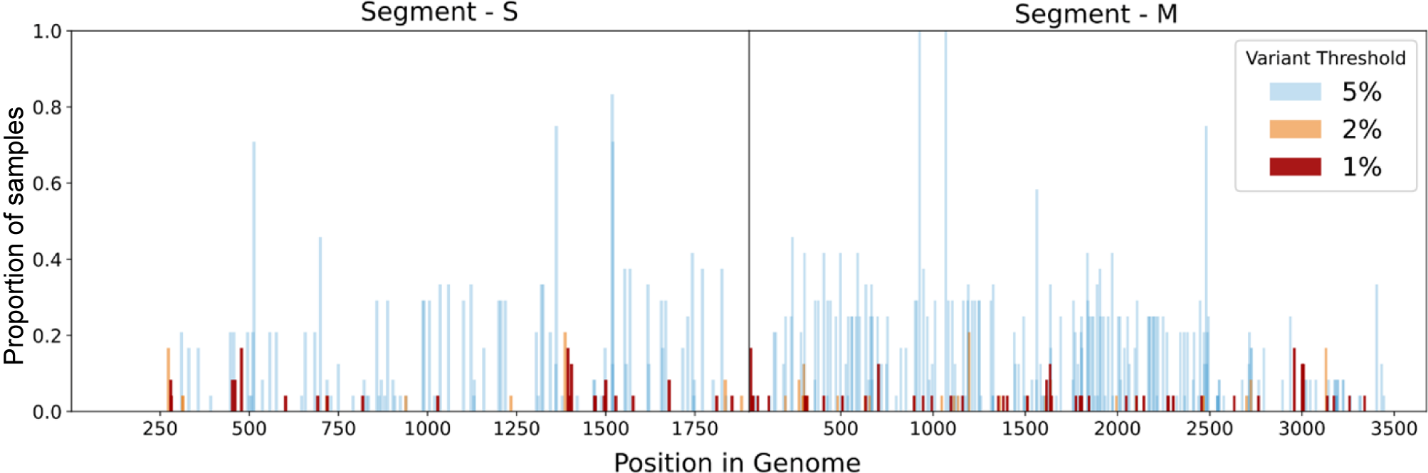
 S2 Figure.** **Distribution of unique single-nucleotide polymorphisms in the S- and M-segment genomes**. The unique single-nucleotide polymorphisms (SNPs) are colored by the significance threshold at which they were identified (1% (red), 2% (orange), or 5% (black)), with lower thresholds capturing rarer mutations. The x-axis represents position in the S- or M-segment vRNA, while the y-axis indicates SNP frequency.
