# Supplementary material for: Purifying selection constrains the evolution of Juquitiba virus in wild Oligoryzomys nigripes communities
Source: PLoS Pathog. 2026 Jan 20;22(1):e1013839. doi: 10.1371/journal.ppat.1013839 (PMC12844527; doi:10.1371/journal.ppat.1013839)
Supplement: S3 Fig — (DOCX) [file ppat.1013839.s003.docx]

**
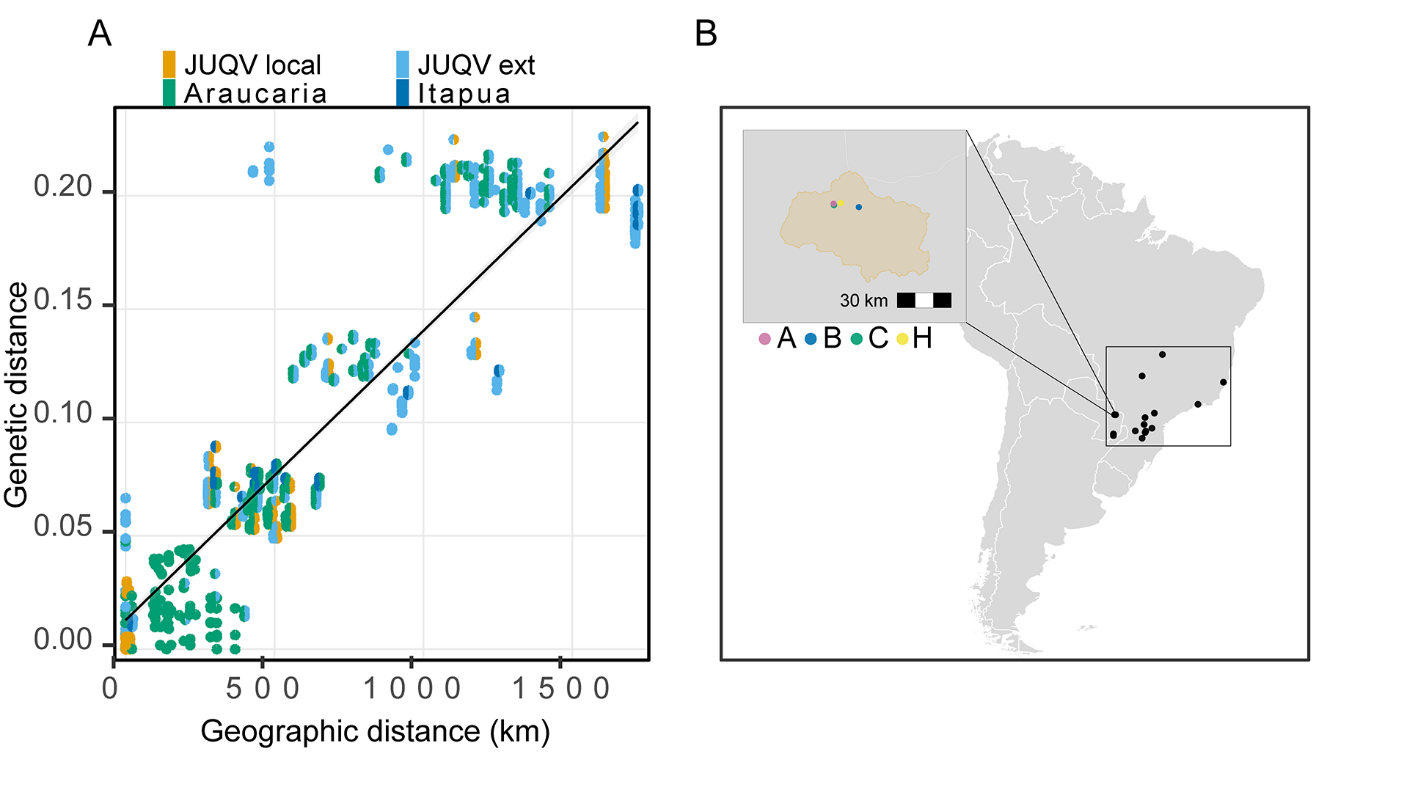
S3 Figure.** (**A**) Patristic analysis of JUQV and ARAV nucleotide sequences and (**B**) map showing locations of sequenced samples. Basemap generated from the Natural Earth “Admin 0 – Countries” shapefile (1:10m scale; Public Domain/CC0; https://www.naturalearthdata.com/http//www.naturalearthdata.com/download/10m/cultural/ne_10m_admin_0_countries.zip). Protected area boundaries derived from the World Database on Protected Areas (WDPA, Paraguay dataset), accessed via the wdpar R package.
