# Supplementary material for: Purifying selection constrains the evolution of Juquitiba virus in wild Oligoryzomys nigripes communities
Source: PLoS Pathog. 2026 Jan 20;22(1):e1013839. doi: 10.1371/journal.ppat.1013839 (PMC12844527; doi:10.1371/journal.ppat.1013839)
Supplement: S4 Fig — (DOCX) [file ppat.1013839.s004.docx]

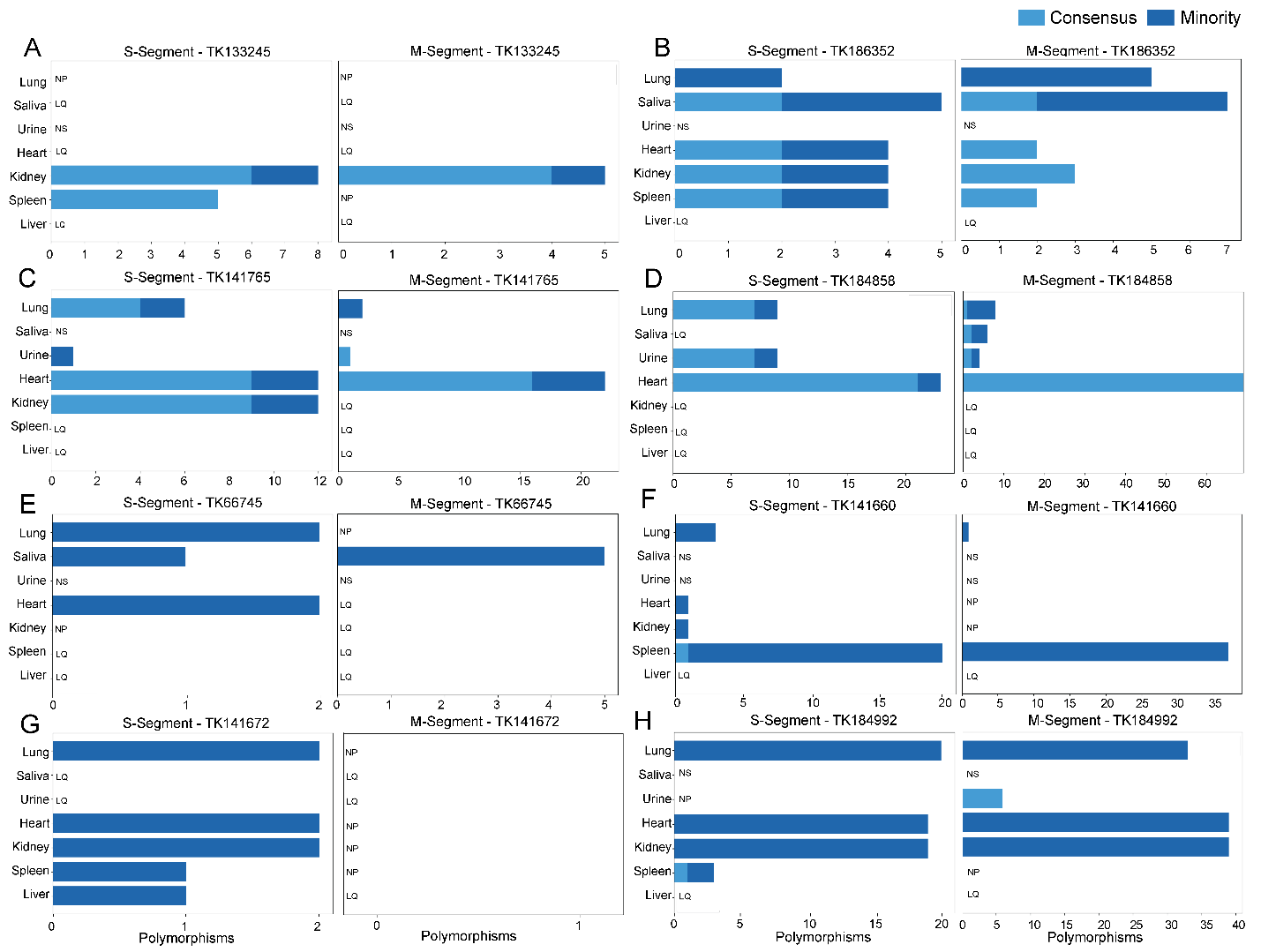
 **S4 Figure.** **Number of consensus and minority polymorphisms in vRNA genomes from tissues and excreta of individual Oligoryzomys rodents.** Consensus polymorphisms (≥50% frequency, x-axis) are shown in light blue, and minority polymorphisms (<50% frequency) in dark blue. The y-axis indicates sample type. Samples are labeled as not screened (NS), low quality (LQ; did not meet the ≥80% genome coverage and ≥500× depth criteria), or containing no polymorphisms (NP). vRNA screening results are shown in **Fig. 1**. Samples meeting inclusion criteria were: TK133245: lung, heart, kidney, spleen; TK186352: lung, saliva, heart, kidney, spleen; liver (S-segment only); TK141765: lung, urine, heart (both segments); kidney (S-segment only); TK184858: lung, urine, heart (both segments); saliva (M-segment only); TK66745: lung, saliva, heart, kidney (S-segment); lung, saliva (M-segment); TK141660: lung, heart, kidney, spleen; TK141672: lung, heart, kidney, spleen; liver (S-segment only); TK184992: lung, urine, heart, kidney, spleen.
