# Supplementary material for: Purifying selection constrains the evolution of Juquitiba virus in wild Oligoryzomys nigripes communities
Source: PLoS Pathog. 2026 Jan 20;22(1):e1013839. doi: 10.1371/journal.ppat.1013839 (PMC12844527; doi:10.1371/journal.ppat.1013839)
Supplement: S1 Table — (DOCX) [file ppat.1013839.s005.docx]

**S1 Table.** **JUQV forward primers for the S-, M-, and L-segments are listed along with their associated primer pools**

| **Name** | **Region** | **5´-3´ Sequence** | **Length (bp)** | **GC Content** | **Melting Tm** | **Pool** |
| --- | --- | --- | --- | --- | --- | --- |
| SF1 | 1-32F | TAGTAGTAGACTCCTTGAGAAGCTAC | 32 | 40.6% | 65.8°C | 1 and 4 |
| SF2 | 262-287F | CTGGCTACAAAACCAGTTGATCCAAC | 26 | 46.2% | 66.2°C | 2 |
| SF3 | 454-477F | CTGTACATGCTTTCAACCCGAGGG | 24 | 54.2% | 67.1°C | 3 |
| SF4 | 687-717F | CATAAGTCCTGTMATGGGAGTCATTGGATTC | 31 | 43.5% | 67.4°C | 1 |
| SF5 | 910-938F | GAATCTGCAACATTGTTTACTGACATTGC | 29 | 37.9% | 66.0°C | 2 and 5 |
| SF6 | 1126-1157F | CAATCYTACTTGAGAAGAACTCAATCTATGGG | 32 | 39.1% | 65.2°C | 3 |
| SF7 | 1368-1395F | GCTTGAGCCAATCAGTTAYTCATCTCAG | 28 | 44.6% | 66.4ºC | 1 |
| SF8 | 1469-1497F | GGTTTATGGGCCACTACTGTTCTTATTTG | 30 | 41.4% | 66.1ºC | 2 |
| MF1 | 1-32F | TAGTAGTAGACTCCGCAAGAAGAAGCAAAAAC | 32 | 40.6% | 68.0°C | 1 and 4 |
| MF2 | 271-300F | CTTCAAAAGAGCTACACACAGGTAGAATGG | 30 | 43.3% | 67.0°C | 2 |
| MF3 | 451-478F | GATTTAACATGTAATCAGACCCACTGCC | 28 | 42.9% | 66.0°C | 3 |
| MF4 | 715-747F | GACCAACTTAAGATAATTAGTACATTTGAGGGC | 33 | 36.4% | 66.0°C | 1 |
| MF5 | 872-898F | GTCGTATGATAGTACACCCCAGAGGTG | 27 | 51.9% | 67.0°C | 2 |
| MF6 | 1171-1197F | GAAAAGGTCACTGGCTGCACAGTATTC | 27 | 48.1% | 67.6°C | 3 |
| MF7 | 1357-1391F | GTTTATTGTAATGGTCAGAAGAAAGTTATCTTGAC | 35 | 31.4% | 65.5ºC | 1 |
| MF8 | 1604-1635F | CATGCTCCCATTACACTAATGAGTCAAAATTC | 32 | 37.5% | 66.6ºC | 2 |
| MF9 | 1770-1801F | CCCATATTGTATGACCATGACTGAATCTACAG | 32 | 40.6% | 66.5ºC | 3 |
| MF10 | 2064-2097F | CATGAAAACAGATTTGGAGCTAGATTTCTCATTG | 34 | 35.3% | 66.9°C | 1 |
| MF11 | 2244-2271F | CATAAAGACTGCCTTTCATTGTTACGGG | 28 | 42.9% | 66.2ºC | 2 |
| MF12 | 2519-2545F | CAAATGACTGCCTTGTAACACCTTCTG | 27 | 44.4% | 66.3ºC | 3 |
| MF13 | 2671-2699F | GATCCCGGTGATATAATGTCTACAACTGC | 29 | 44.8% | 66.5°C | 1 and 5 |
| MF14 | 2946-2977F | GGATCTAAGTGATAATCCATGTAAGGTAGACC | 32 | 40.6% | 65.8°C | 2 |
| MF15 | 3127-3153F | GCAAGGGGCTCTAATACAGTCAAAGTG | 27 | 48.1% | 67.1°C | 3 |
| LF1 | 1-31F | TAGTAGTAGACTCCGGGATAGAAAAADTCAG | 31 | 39.8% | 65.4°C | 1 and 3 |
| LF2 | 274-301F | GATAATYCCTAACAGYYCTTCAGGACAG | 28 | 45.0% | 65.1°C | 2 |
| LF3 | 507-536F | GTAGTAGCTGTTAAGACAGATGGGTCTAAC | 30 | 43.3% | 65.9°C | 3 |
| LF4 | 788-819F | GACAGAAGAGCATAAGTTTGTATTTAAGGAGG | 32 | 37.5% | 65.4ºC | 1 |
| LF5 | 1113-1141F | CAAWCAAGTYCAAATGTWAAAGARCCTGGC | 29 | 40.0% | 65.6ºC | 2 |
| LF6 | 1596-1629F | GAAGTAGTTGGCTCTTACATTAGGTATTTTACAG | 34 | 35.3% | 65.7°C | 3 |
| LF7 | 1786-1818F | CATGGTTTCAGTATTATACAGAAGATCAAGGTC | 33 | 35.3% | 65.7°C | 1 |
| LF8 | 1925-1959F | GATTCCTTCTGTTACTTCATTATATTCAGGATATG | 35 | 31.4% | 64.7°C | 2 |
| LF9 | 2160-2195F | GTTGAGTACAAACATTATAGGAGCTTAATATCAGAG | 36 | 33.3% | 65.8°C | 3 |
| LF10 | 2393-2419F | CTGYCAAGAGGTGTCAGARTTRTGTG | 26 | 48.1% | 66.2ºC | 1 |

**S1 Table. Continued**

| **Name** | **Region** | **5´-3´ Sequence** | **Length (bp)** | **GC Content** | **Melting Tm** | **Pool** |
| --- | --- | --- | --- | --- | --- | --- |
| LF11 | 2740-2778F | CAAGAGTTCGACTAGAAATAATAGAGGATTATTTTGATG | 39 | 30.8% | 66.3°C | 2 |
| LF12 | 2983-3009F | CAGCAAAGTTTCGGAGGTTTACACAAG | 27 | 44.4% | 66.5ºC | 3 |
| LF13 | 3122-3150F | CAATATGGATGGTCATTCAGAAGCTGTTG | 29 | 41.4% | 66.3ºC | 1 |
| LF14 | 3495-3526F | GGCTCYATTAAAGTATCACCRAAAAAGACTAC | 32 | 38.0% | 66.0°C | 2 |
| LF15 | 3807-3834F | CAGAARTCCCATGTCCCRATACCATTAG | 28 | 46.4% | 66.6ºC | 3 |
| LF16 | 4044-4072F | GTRCAATGGAAGGTTTTCACACCTAAATC | 29 | 39.7% | 65.9ºC | 1 |
| LF17 | 4347-4373F | GCAACCAATTATCAACCAACRGAAAGG | 27 | 42.6% | 66.2ºC | 2 |
| LF18 | 4620-4647F | CCAGATTCACTATCTGCTGATCTRCAAG | 28 | 44.6% | 65.8ºC | 3 |
| LF19 | 4900-4926F | GATTACAGGATGATATTGCYGCYACAC | 27 | 44.4% | 65.7ºC | 1 |
| LF20 | 5180-5214F | GATGCTTATTGTRTTCAAACTTATAATGAAGAGAC | 35 | 30.0% | 65.1ºC | 2 |
| LF21 | 5474-5506F | GTATAARCATTGTGTTCTACGAACAGGRTTRAG | 33 | 37.9% | 66.7°C | 3 |
| LF21 | 5658-5687F | GTAGGGAATGTTTGGTTCAAAACAGAACAG | 28 | 44.6% | 66.3ºC | 1 |
| LF23 | 5942-5975F | GATGAGTTTAATGTCRGAAGATGATATTGATGAC | 34 | 33.8% | 65.6ºC | 2 |
| LF24 | 5994-6026F | GCATTAGATGACATTGACTTTGAGCAGATTAAC | 33 | 36.4% | 66.8ºC | 3 |

The forward primer sequences are directionally antigenomic and are in reference to complementary RNA (cRNA). These primers bind to and amplify S, M, or L segment vRNA. The primer name is designated by segment (S, M, or L), primer type (F=forward) and specific number ID (1-24).
