# Supplementary material for: Purifying selection constrains the evolution of Juquitiba virus in wild Oligoryzomys nigripes communities
Source: PLoS Pathog. 2026 Jan 20;22(1):e1013839. doi: 10.1371/journal.ppat.1013839 (PMC12844527; doi:10.1371/journal.ppat.1013839)
Supplement: S2 Table — (DOCX) [file ppat.1013839.s006.docx]

**S2 Table.** **JUQV reverse primers for the S-, M-, and L-segments are listed along with their associated primer pools**

| **Name** | **Region** | **5´-3´ Sequence** | **Length (bp)** | **GC Content** | **Melting Tm** | **Pool** |
| --- | --- | --- | --- | --- | --- | --- |
| SR1 | 503-534R | GGATGAGTCATCTTTAAACCTRATYCTAGTCC | 32 | 40.6% | 66.3°C | 1 |
| SR2 | 800-828R | CTTATTYGTTGCCATGAATGCCTCAGATG | 29 | 43.1% | 67.3°C | 2 |
| SR3 | 938-965R | GCAAATACCCATACAGAAKGTGGTGTTG | 28 | 44.6% | 67.0°C | 3 and 4 |
| SR4 | 1235-1260R | CCTGAGTTCTGGGTCCATATCATCTC | 26 | 50.0% | 65.7°C | 1 |
| SR5 | 1388-1421R | CTTAGTAGTCACATTAAGGAAAGATCCTGAGATG | 34 | 38.2% | 66.3°C | 2 |
| SR6 | 1622-1656R | CCTACATACACTYAATATAATAATTCTGGCCAATC | 35 | 32.9% | 65.4°C | 3 |
| SR7 | 1833-1867R | GCATTATTGAGGTAGTATTGTTGAGGTAGTTAAGG | 35 | 37.9% | 66.9ºC | 1 |
| SR8 | 1873-1902R | TAGTAGTATGCTCCTTGAAAAGCAATCAAG | 30 | 36.7% | 65.7ºC | 2 and 5 |
| MR1 | 515-546R | CATACTAACCATACAGCTCCGTATTGACATAC | 32 | 40.6% | 66.4°C | 1 |
| MR2 | 787-820R | CCGAGTGACTTCCTAAAAAACAGACATAATATCC | 34 | 38.2% | 67.0°C | 2 |
| MR3 | 974-1004R | GCAGTACCTTTCATAGTGTCTGTTGAACTAG | 31 | 41.9% | 66.6°C | 3 and 4 |
| MR4 | 1240-1271R | GTAGGAGAGCTGATGTTAAAGATACCATTCTC | 32 | 40.6% | 66.2°C | 1 |
| MR5 | 1391-1415R | CATTGCCCAATCACAAGGGTCTTTG | 25 | 48.0% | 66.7°C | 2 |
| MR6 | 1644-1676R | GTCTTCTGGTACTCTACTTTAACTTTCTCTAGG | 33 | 39.4% | 65.7°C | 3 |
| MR7 | 1883-1910R | GTACGGTAGCAACCTCTTTTGATCTCTG | 28 | 46.4% | 66.6°C | 1 |
| MR8 | 2105-2135R | GTTAATTTCCGCCTATAGCTGTAAGATGATG | 31 | 38.7% | 65.8ºC | 2 |
| MR9 | 2272-2299R | GCCAAGGGTATGAATACTTTTGACATGC | 28 | 42.9% | 66.6ºC | 3 |
| MR10 | 2543-2573R | GAAACTGTCCCTATAATGCAGACCTTTACAG | 31 | 41.9% | 66.8°C | 1 |
| MR11 | 2766-2794R | CGGATACAGTATTGCCCTGATATTCACAC | 29 | 44.8% | 66.7ºC | 2 |
| MR12 | 3050-3082R | CCTTGATTGATGTAATGAAACTTGGACATTCTG | 33 | 36.4% | 66.8ºC | 3 |
| MR13 | 3169-3196R | GGCAACACTTAAAAGAAGAACCTGAATG | 28 | 39.3% | 65.3°C | 1 |
| MR14 | 3453-3480R | GCCCATCGGAGATTAAACAGTTTTCTTG | 28 | 42.9% | 66.4°C | 2 |
| MR15 | 3647-3674R | TAGTAGTATGCTCGCAGGAACAAAATCC | 28 | 42.9% | 66.6°C | 3 and 5 |
| LR1 | 560-582R | GTTGTACTACACCATCATTCCTCCTACTAG | 30 | 43.3% | 65.6°C | 1 |
| LR2 | 343-376R | GTAACAGTCACCTCAATAAATTCAATYAGRTTGC | 34 | 35.3% | 66.9°C | 3 |
| LR3 | 747-781R | CCTTTACAATAATTTAGYAGGTTCTCAATTCGTGG | 35 | 35.7% | 66.7°C | 2 |
| LR4 | 1048-1073R | CTTCGTTAGGCTCAGGTACTTGTATG | 26 | 46.2% | 64.7°C | 3 |
| LR5 | 1299-1332R | CAASATTGATTTCAAGGTTAGAYAAGATTTGCTC | 34 | 33.8% | 66.1°C | 1 |
| LR6 | 1593-1626R | CTGTAAAATACCTAATGTAAGAGCCAACTACTTC | 34 | 35.3% | 65.7°C | 2 |
| LR7 | 2144-2173R | GTTTGTACTCAACCCGTGACATTAAAGAG | 29 | 41.4% | 66.0°C | 3 |
| LR8 | 2357-2387R | CTACTTGAACCTCACCATTGRTTATTGACTC | 31 | 40.0% | 66.3°C | 1 |
| LR9 | 2477-2508R | GACTAAAATAGGGTTTATCCCAATGCTTATTC | 32 | 34.4% | 65.0°C | 2 |
| LR10 | 2653-2682R | CAATCCTAGCTTGTGCTTTAAACTGTTTAG | 30 | 37.0% | 65.2°C | 3 |

**S2 Table. Continued**

| **Name** | **Region** | **5´-3´ Sequence** | **Length (bp)** | **GC Content** | **Melting Tm** | **Pool** |
| --- | --- | --- | --- | --- | --- | --- |
| LR11 | 2880-2908R | CCTGTACTTGTRACAATTTCAGAAATCCC | 29 | 39.7% | 66.5ºC | 1 |
| LR12 | 3270-3297R | GCTCAGGATAAAGTTGYTTCCAGATTTC | 28 | 42.9% | 65.5ºC | 2 |
| LR13 | 3444-3474R | GTAGGTTAAACATACTTTTCCACATCTCYTG | 31 | 37.1% | 65.2°C | 3 |
| LR14 | 3615-3641R | GAARCCTAACCCAGGCARATCTKAAAG | 27 | 46.3% | 66.5°C | 1 |
| LR15 | 3980-4008R | GCTGAAAGACATCCTCACTTAACKACMTC | 29 | 44.8% | 66.7°C | 2 |
| LR16 | 4266-4298R | CTCATATCTACATACCTTCATATGCTGTTTRGC | 33 | 37.9% | 66.2°C | 3 |
| LR17 | 4486-4516R | CTTACAGTAAATGTTCGTGCAATCTTTGGTC | 31 | 38.7% | 66.7°C | 1 |
| LR18 | 4839-4867R | GCCTTCAATACTTCCTTCTTTGGTCTCAC | 29 | 45.0% | 67.1°C | 2 |
| LR19 | 5096-5127R | GCCTGCATCTGAAGCACACTTTTATAAC | 32 | 37.5% | 66.4°C | 3 |
| LR20 | 5407-5434R | GCYTCTGTTTTGAACAAGACAAGACTTC | 28 | 41.1% | 65.9ºC | 1 |
| LR21 | 5706-5741R | CATAAAGTTTTCTAGCAATCCTGAAGAGATAATTGG | 36 | 33.0% | 66.6°C | 2 |
| LR22 | 5942-5975R | GTCATCAATATCATCTTCYGACATTAAACTCATC | 34 | 33.8% | 65.6ºC | 3 |
| LR23 | 6189-6226R | GCCATAAGAATAATGCCTACAGGGTTATATACTTTATC | 38 | 34.2% | 67.0°C | 1 |
| LR24 | 6417-6447R | CATCMACATGCCTGAATGAAAATAAGGAATC | 31 | 37.1% | 66.3°C | 2 |
| LR25 | 6535-6564R | TAGTAGTATGCTCCGGGAAAAGAACACTAC | 30 | 43.3% | 67.2°C | 3 |

The reverse primer sequences are directionally genomic and are in reference to viral RNA (vRNA). These primers bind to and amplify S, M, or L segment complementary or antigenomic RNA (cRNA). The primer name is designated by segment (S, M, or L), primer type (F=forward) and specific number ID (1-25).
