# Supplementary material for: Purifying selection constrains the evolution of Juquitiba virus in wild Oligoryzomys nigripes communities
Source: PLoS Pathog. 2026 Jan 20;22(1):e1013839. doi: 10.1371/journal.ppat.1013839 (PMC12844527; doi:10.1371/journal.ppat.1013839)
Supplement: S3 Table — (DOCX) [file ppat.1013839.s007.docx]

S3 Table. *Oligoryzomys spp*. samples used in this study

| Sampling Year | Lung | **Heart** | **Spleen** | **Kidney** | **Liver** | **Saliva** | **Urine** |
| --- | --- | --- | --- | --- | --- | --- | --- |
| 2014 | 8 | - | - | - | - | 1 | - |
| 2015 | - | - | - | - | - | 9 | 1 |
| 2016 | 55 | 4 | 4 | 4 | 4 | 15 | 7 |
| 2017 | 36 | 4 | 4 | 4 | 4 | 6 | 4 |
| Total | 99 | 8 | 8 | 8 | 8 | 31 | 12 |

Values designated by a dashed line (-) indicate that no sample was tested or available for that collection year.
