# Supplementary material for: Purifying selection constrains the evolution of Juquitiba virus in wild Oligoryzomys nigripes communities
Source: PLoS Pathog. 2026 Jan 20;22(1):e1013839. doi: 10.1371/journal.ppat.1013839 (PMC12844527; doi:10.1371/journal.ppat.1013839)
Supplement: S4 Table — (DOCX) [file ppat.1013839.s008.docx]

**S4 Table. JUQV GenBank Accession numbers**

| **Sample ID** | **Sample Type** | **S Segment Genbank Accesion No.** | **M Segment Genbank Accesion No.** |
| --- | --- | --- | --- |
| TK132709 | Lung | OR184962 | OR184978 |
| TK133233 | Saliva | PX395964 | PX395975 |
| TK133245 | Lung | OR184963 | OR184979 |
| TK133245 | Liver | PX395965 | PV052297 |
| TK133245 | Saliva | PX395966 | PX395983 |
| TK133245 | Spleen | PV007716 | PX395981 |
| TK133245 | Heart | PV007714 | PV052295 |
| TK133245 | Kidney | PV007715 | PV052296 |
| TK141528 | Saliva | PV007744 | PV052319 |
| TK141638 | Lung | OR184964 | OR184980 |
| TK141660 | Lung | OR184965 | OR184981 |
| TK141660 | Heart | PV007717 | PV052298 |
| TK141660 | Kidney | PV007718 | PV052299 |
| TK141660 | Liver | PV007719 | PV052300 |
| TK141660 | Spleen | PV007720 | PX422820 |
| TK141672 | Lung | OR184966 | OR184982 |
| TK141672 | Saliva | PX395967 | PX395978 |
| TK141672 | Urine | PX395968 | PX395980 |
| TK141672 | Spleen | PV007724 | PX395979 |
| TK141672 | Heart | PV007721 | PV052301 |
| TK141672 | Kidney | PV007722 | PV052302 |
| TK141672 | Liver | PV007723 | PV052303 |
| TK141765 | Lung | OR184967 | OR184983 |
| TK141765 | Urine | PX395969 | PX395977 |
| TK141765 | Spleen | PV007728 | PX395976 |
| TK141765 | Heart | PV007725 | PV052304 |
| TK141765 | Kidney | PV007726 | PV052305 |
| TK141765 | Liver | PV007727 | PV052306 |
| TK170224 | Saliva | PV007745 | PV052320 |
| TK170226 | Saliva | PX395970 | PX395982 |
| TK184781 | Urine | PV007746 | PV052321 |
| TK184858 | Lung | OR184968 | OR184984 |
| TK184858 | Spleen | PV007732 | PX395984 |
| TK184858 | Heart | PV007729 | PV052307 |
| TK184858 | Kidney | PV007730 | PV052308 |
| TK184858 | Liver | PV007731 | PV052309 |
| TK184858 | Saliva | PV007747 | PV052322 |
| TK184858 | Urine | PV007748 | PV052323 |
| TK184889 | Lung | OR184975 | OR184985 |
| TK184889 | Saliva | PV007749 | PV052324 |
| TK184992 | Lung | OR184959 | OR184986 |
| TK184992 | Heart | PV007733 | PV052310 |
| TK184992 | Kidney | PV007734 | PV052311 |
| TK184992 | Liver | PV007735 | PV052312 |
| TK184992 | Spleen | PV007736 | PX422821 |
| TK184992 | Urine | PV007750 | PV052325 |
| TK186283 | Lung | OR184969 | OR184987 |
| TK186283 | Saliva | PV007751 | PV052326 |
| TK186318 | Lung | OR184970 | OR184988 |
| TK186352 | Lung | OR184971 | OR184989 |
| TK186352 | Spleen | PV007740 | PX395985 |
| TK186352 | Heart | PV007737 | PV052313 |
| TK186352 | Kidney | PV007738 | PV052314 |
| TK186352 | Liver | PV007739 | PV052315 |
| TK186352 | Saliva | PV007752 | PV052327 |
| TK186353 | Lung | OR184972 | OR184990 |
| TK246023 | Lung | OR184973 | OR184991 |
| TK246028 | Saliva | PX395971 | PX395986 |
| TK246099 | Lung | OR184974 | OR184992 |
| TK246099 | Saliva | PX395972 | PX395987 |
| TK66695 | Lung | OR184960 | OR184976 |
| TK66695 | Urine | PX395973 | * |
| TK66745 | Lung | OR184961 | OR184977 |
| TK66745 | Liver | PX395974 | PV052318 |
| TK66745 | Spleen | PV007743 | PX395988 |
| TK66745 | Heart | PV007741 | PV052316 |
| TK66745 | Kidney | PV007742 | PV052317 |
| TK66745 | Saliva | PV007753 | PV052328 |

* GenBank accession number is not available for the TK66695 M-segment (urine) sequence because the sample yielded too few reads at low coverage to generate a consensus.
