# Supplementary material for: Purifying selection constrains the evolution of Juquitiba virus in wild Oligoryzomys nigripes communities
Source: PLoS Pathog. 2026 Jan 20;22(1):e1013839. doi: 10.1371/journal.ppat.1013839 (PMC12844527; doi:10.1371/journal.ppat.1013839)
Supplement: S5 Table — (DOCX) [file ppat.1013839.s009.docx]

**S5 Table.** **Genome coverage and average depth of coverage of JUQV S- and M-segment vRNA from *Oligoryzomys* lungs**

| **Grid** | **TK** | **Total**  **Reads** | **Reads**  **Mapped** |  | **S segment** | | | |  | **M segment** | | | |
| --- | --- | --- | --- | --- | --- | --- | --- | --- | --- | --- | --- | --- | --- |
|  |  |  |  |  | **Total Read**  **Count** | **% Total** | **Depth** | **Coverage** |  | **Total Read**  **Count** | **% Total** | **Depth** | **Coverage** |
| A | TK184889 | 2,268,760 | 2,208,865 |  | 427,084 | 19.30% | 16,625 | 100% |  | 1,657,064 | 75.00% | 33,366 | 100% |
|  | TK246099 | 3,357,484 | 3,229,900 |  | 896,552 | 27.80% | 34,989 | 100% |  | 2,026,348 | 62.70% | 41,018 | 97% |
| B | TK133245 | 1,962,660 | 1,923,014 |  | 5,193 | 0.30% | 192.07 | 96% |  | 14,997 | 0.80% | 291.64 | 94% |
|  | TK186318 | 1,672,878 | 1,636,577 |  | 95,293 | 5.80% | 3,758 | 99% |  | 91,542 | 5.60% | 1,860 | 94% |
|  | TK186352 | 712,180 | 611,834 |  | 66,533 | 10.90% | 2,594 | 98% |  | 180,111 | 29.40% | 3,648 | 95% |
|  | TK186353 | 2,691,564 | 2,606,241 |  | 470,762 | 18.10% | 18,318 | 100% |  | 1,972,732 | 75.70% | 39,902 | 100% |
| C | TK66695 | 1,024,518 | 963,969 |  | 304,591 | 31.60% | 11,899 | 100% |  | 407,815 | 42.30% | 8,282 | 99% |
|  | TK66745* | 2,451,512 | 2,388,998 |  | 400,390 | 16.80% | 15,626 | 99% |  | 1,870,653 | 78.30% | 38,661 | 100% |
|  | TK132709 | 1,752,118 | 1,691,320 |  | 357,607 | 21.10% | 14,033 | 100% |  | 992,947 | 58.70% | 20,219 | 99% |
|  | TK141638 | 3,087,394 | 2,962,046 |  | 1,172,980 | 39.60% | 45,646 | 100% |  | 1,646,553 | 55.60% | 33,295 | 99% |
|  | TK141660 | 2,247,600 | 2,166,911 |  | 437,343 | 20.20% | 17,033 | 99% |  | 1,539,720 | 71.10% | 31,173 | 99% |
|  | TK141672 | 3,148,010 | 3,030,274 |  | 1,289,324 | 42.50% | 50,208 | 100% |  | 1,474,828 | 48.70% | 29,800 | 99% |
|  | TK184992 | 3,470,046 | 3,329,509 |  | 940,690 | 28.30% | 36,602 | 100% |  | 2,130,226 | 64.00% | 43,038 | 100% |
|  | TK186283 | 1,076,020 | 969,386 |  | 273,444 | 28.20% | 10,644 | 100% |  | 361,188 | 37.30% | 7,300 | 98% |
|  | TK246023 | 1,287,440 | 1,278,685 |  | 350,284 | 27.40% | 13,845 | 100% |  | 596,124 | 46.60% | 12,179 | 100% |
| H | TK141765 | 660,342 | 554,621 |  | 154,139 | 27.80% | 5,942 | 100% |  | 59,547 | 10.70% | 1,187 | 98% |
|  | TK184858 | 1,825,650 | 1,678,685 |  | 663,176 | 39.50% | 25,708 | 100% |  | 503,410 | 30.00% | 10,111 | 96% |

Grid indicates the location each animal was collected from. Detailed grid information can be found in the supplemental text of Camp et al. 2021 [[1](#_ENREF_1)]. TK = the rodent identification number. A star (*) indicates that the sequence was obtained from *Oligoryzomys mattogrossae*. All other sequences were obtained from *O. nigripes*. Sequences from TK184992 were used as references for read mapping.
