# Supplementary material for: Purifying selection constrains the evolution of Juquitiba virus in wild Oligoryzomys nigripes communities
Source: PLoS Pathog. 2026 Jan 20;22(1):e1013839. doi: 10.1371/journal.ppat.1013839 (PMC12844527; doi:10.1371/journal.ppat.1013839)
Supplement: S6 Table — (DOCX) [file ppat.1013839.s010.docx]

S6 Table. Genome coverage and average depth of coverage of JUQV S- and M-segment vRNA from *Oligoryzomys* saliva and urine

| **Grid** | **TK** | **Sample** | **Reads**  **Mapped** |  | **S Segment** | | | |  | | **M Segment** | | | | |  |
| --- | --- | --- | --- | --- | --- | --- | --- | --- | --- | --- | --- | --- | --- | --- | --- | --- |
|  |  |  |  |  | **Total Read**  **Count** | **% Total** | **Depth** | **Coverage** | |  | | **Total Read**  **Count** | **% Total** | **Depth** | **Coverage** | |
| A | TK184889 | Saliva | 1,439,820 |  | 64,810 | 4.50% | 2,327.88 | 86% | |  | | 407,711 | 28.3% | 8,189.77 | 100% | |
|  | TK246099 | Saliva | 2,353,054 |  | 11,866 | 0.50% | 323.14 | 84% | |  | | 49,508 | 2.1% | 877.04 | 79% | |
| B | TK133245 | Saliva | 1,740,110 |  | 20,109 | 1.16% | 391.31 | 69% | |  | | 10,183 | 0.59% | 84.53 | 60% | |
|  | TK184781 | Urine | 2,111,568 |  | 136,311 | 6.46% | 5,052.12 | 86% | |  | | 174,845 | 8.28% | 3,025.12 | 88% | |
|  | TK186352 | Saliva | 2,231,610 |  | 126,154 | 5.65% | 4,506.70 | 86% | |  | | 279,453 | 12.5% | 5,530.24 | 100% | |
| C | TK66695 | Urine | 1,487,478 |  | 25,647 | 1.72% | 661.85 | 55% | |  | | 40,685 | 2.7% | 318.32 | 49% | |
|  | TK66745^*^ | Saliva | 1,812,758 |  | 53,913 | 2.97% | 2,105.61 | 86% | |  | | 574,329 | 31.6% | 11,662.1 | 100% | |
|  | TK141672 | Saliva | 2,229,628 |  | 26,004 | 1.17% | 926.89 | 45% | |  | | 82,749 | 3.71% | 1,509.13 | 61% | |
|  | TK141672 | Urine | 2,149,712 |  | 31,623 | 1.47% | 518.22 | 43% | |  | | 152,178 | 7.08% | 2,129.23 | 56% | |
|  | TK186283 | Saliva | 1,649,610 |  | 86,216 | 5.23% | 3,179.26 | 90% | |  | | 110,288 | 6.6% | 2,161.22 | 90% | |
|  | TK184992 | Urine | 2,120,554 |  | 23,625 | 1.11% | 698.71 | 84% | |  | | 261,974 | 12.4% | 4,471.81 | 95% | |
|  | TK246028 | Saliva | 2,599,710 |  | 9,966 | 0.38% | 134.15 | 80% | |  | | 33,153 | 1.28% | 252.69 | 94% | |
| D | TK133233 | Saliva | 1,835,826 |  | 3,570 | 0.19% | 48.13 | 73% | |  | | 11,112 | 0.61% | 85.23 | 89% | |
| G | TK141528 | Saliva | 3,876,160 |  | 78,121 | 2.02% | 2,859.76 | 80% | |  | | 61,648 | 1.59% | 1,021.08 | 92% | |
|  | TK170224 | Saliva | 3,911,722 |  | 70,891 | 1.81% | 2,641.77 | 82% | |  | | 730,472 | 18.7% | 1,170.00 | 96% | |
|  | TK170226 | Saliva | 1,162,010 |  | 3,216 | 0.28% | 56.03 | 69% | |  | | 18,244 | 1.57% | 250.87 | 74% | |
| H | TK141765 | Urine | 1,821,364 |  | 29,696 | 1.63% | 1,035.84 | 84% | |  | | 167,386 | 9.19% | 3,073.96 | 99% | |
|  | TK184858 | Urine | 1,735,650 |  | 131,590 | 7.58% | 4,913.46 | 83% | |  | | 432,422 | 24.9% | 8,283.27 | 100% | |
|  | TK184858 | Saliva | 1,266,184 |  | 49,719 | 3.93% | 1,857.98 | 78% | |  | | 401,300 | 31.7% | 7,953.22 | 84% | |

Grid indicates the location each animal was collected from. Detailed grid information can be found in the supplemental text of Camp et al. 2021 [1]. TK indicates the rodent identification number. When TK is followed by a star (*) this indicated that the sequence was obtained from *Oligoryzomys mattogrossae*. All other sequences were isolated from *O. nigripes*. Sequences from TK184992 were used as the reference sequence.
