# Supplementary material for: Purifying selection constrains the evolution of Juquitiba virus in wild Oligoryzomys nigripes communities
Source: PLoS Pathog. 2026 Jan 20;22(1):e1013839. doi: 10.1371/journal.ppat.1013839 (PMC12844527; doi:10.1371/journal.ppat.1013839)
Supplement: S7 Table — (DOCX) [file ppat.1013839.s011.docx]

S7 Table. Genome coverage and average depth of coverage of JUQV S- and M-segment vRNA from *Oligoryzomys* heart, kidney, spleen, and liver

| **TK** | **Sample** | **Total Reads** | **Reads Mapped** |  | **S Segment** | | | |  | | **M Segment** | | | |
| --- | --- | --- | --- | --- | --- | --- | --- | --- | --- | --- | --- | --- | --- | --- |
|  |  |  |  |  | **Total Read Count** | **% Total** | **Depth** | **Coverage** |  | **Total Read Count** | | **% Total** | **Depth** | **Coverage** |
| TK133245 | Heart | 1,052,700 | 1,039,436.0 |  | 8,368 | 0.8% | 313.4 | 76% |  | 4,931 | | 0.5% | 92.2 | 94% |
|  | Kidney | 1,591,258 | 1,566,752.6 |  | 220,442 | 14.1% | 8257.2 | 77% |  | 258,133 | | 16.5% | 5267.4 | 94% |
|  | Spleen | 902,520 | 867,231.5 |  | 11,730 | 1.4% | 445.6 | 76% |  | 42,627 | | 4.9% | 867.6 | 94% |
|  | Liver | 2,005,520 | 1,975,838.3 |  | 2,055 | 0.1% | 62.2 | 76% |  | 3,206 | | 0.2% | 35.0 | 92% |
| TK186352 | Heart | 1,566,304 | 1,543,592.6 |  | 223,547 | 14.5% | 8863.1 | 85% |  | 28,927 | | 1.9% | 567.4 | 94% |
|  | Kidney | 1,615,596 | 1,595,885.7 |  | 150,858 | 9.5% | 5980.0 | 84% |  | 44,595 | | 2.8% | 888.1 | 94% |
|  | Spleen | 1,234,936 | 1,217,523.4 |  | 92,728 | 7.6% | 3678.5 | 84% |  | 24,266 | | 2.0% | 492.1 | 94% |
|  | Liver | 1,147,054 | 1,124,915.9 |  | 7,588 | 0.7% | 295.3 | 83% |  | 4,824 | | 0.4% | 84.0 | 94% |
| TK141660 | Heart | 861,572 | 847,959.2 |  | 196,519 | 23.2% | 7763.2 | 96% |  | 178,072 | | 21.0% | 3636.6 | 95% |
|  | Kidney | 2,404,340 | 2,339,182.4 |  | 68,140 | 2.9% | 2667.4 | 95% |  | 43,202 | | 1.8% | 852.2 | 94% |
|  | Spleen | 2,035,028 | 2,000,839.5 |  | 261,218 | 13.1% | 10338.0 | 96% |  | 847,324 | | 42.3% | 17371.5 | 95% |
|  | Liver | 2,406,640 | 2,337,569.4 |  | 1,468 | 0.1% | 33.3 | 84% |  | 3,673 | | 0.2% | 39.5 | 89% |
| TK141672 | Heart | 1,693,216 | 1,663,415.4 |  | 734,728 | 44.2% | 29090.4 | 86% |  | 107,272 | | 6.4% | 2168.8 | 95% |
|  | Kidney | 1,380,304 | 1,353,112.0 |  | 133,306 | 9.9% | 5271.1 | 85% |  | 45,260 | | 3.3% | 897.0 | 94% |
|  | Spleen | 1,347,388 | 1,310,469.6 |  | 289,221 | 22.1% | 11448.3 | 87% |  | 240,788 | | 18.4% | 4926.6 | 95% |
|  | Liver | 1,205,944 | 1,189,422.6 |  | 15,360 | 1.3% | 601.8 | 85% |  | 10,405 | | 0.9% | 191.9 | 94% |
| TK184992 | Heart | 1,542,934 | 1,526,887.5 |  | 446,972 | 29.3% | 17661.2 | 100% |  | 784,905 | | 51.4% | 16048.7 | 100% |
|  | Kidney | 2,035,364 | 2,001,373.4 |  | 241,269 | 12.1% | 9532.0 | 99% |  | 486,637 | | 24.3% | 9943.5 | 95% |
|  | Spleen | 1,982,586 | 1,959,786.3 |  | 204,392 | 10.4% | 8091.6 | 87% |  | 503,046 | | 25.7% | 10310.5 | 97% |
|  | Liver | 2,050,802 | 2,019,219.6 |  | 9,215 | 0.5% | 347.1 | 88% |  | 9,213 | | 0.5% | 173.6 | 94% |

**S7 Table. Continued**

| **TK** | **Sample** | **Total Reads** | **Reads Mapped** |  | **S Segment** | | | |  | **M Segment** | | | |
| --- | --- | --- | --- | --- | --- | --- | --- | --- | --- | --- | --- | --- | --- |
|  |  |  |  |  | **Total Read Count** | **% Total** | **Depth** | **Coverage** |  | **Total Read Count** | **% Total** | **Depth** | **Coverage** |
| TK141765 | Heart | 1,305,222 | 1,288,645.7 |  | 412,588 | 32.0% | 16244.6 | 89% |  | 135,361 | 10.5% | 2761.6 | 94% |
|  | Kidney | 1,444,228 | 1,425,453.0 |  | 83,043 | 5.8% | 3261.6 | 86% |  | 22,891 | 1.6% | 447.5 | 94% |
|  | Spleen | 1,302,118 | 1,285,450.9 |  | 7,018 | 0.5% | 270.7 | 84% |  | 2,628 | 0.2% | 37.7 | 94% |
|  | Liver | 1,261,218 | 1,245,074.4 |  | 4,618 | 0.4% | 176.6 | 85% |  | 2,782 | 0.2% | 40.3 | 92% |
| TK184858 | Heart | 2,197,210 | 2,145,355.8 |  | 174,722 | 8.1% | 6883.3 | 85% |  | 33,333 | 1.6% | 650.0 | 94% |
|  | Kidney | 1,204,064 | 1,186,484.7 |  | 5,994 | 0.5% | 231.3 | 84% |  | 5,521 | 0.5% | 105.1 | 92% |
|  | Spleen | 1,570,496 | 1,515,842.7 |  | 425 | 0.0% | 15.2 | 89% |  | 392 | 0.0% | 7.4 | 89% |
|  | Liver | 1,073,704 | 1,062,537.5 |  | 1,696 | 0.2% | 60.6 | 82% |  | 1,794 | 0.2% | 24.0 | 94% |
| TK66745* | Heart | 1,451,222 | 1,432,936.6 |  | 66,216 | 4.6% | 2618.8 | 84% |  | 16,546 | 1.2% | 315.7 | 94% |
|  | Kidney | 1,260,486 | 1,245,990.4 |  | 20,998 | 1.7% | 828.6 | 84% |  | 10,131 | 0.8% | 186.0 | 94% |
|  | Spleen | 386,964 | 376,090.3 |  | 9,735 | 2.6% | 385.0 | 83% |  | 13,203 | 3.5% | 268.6 | 94% |
|  | Liver | 1,383,258 | 1,365,690.6 |  | 2,108 | 0.2% | 74.2 | 80% |  | 2,818 | 0.2% | 35.2 | 90% |

TK = the rodent identification number. When TK is followed by a star (*) this indicated that the sequence was obtained from *Oligoryzomys mattogrossae*. All other sequences were obtained from *O. nigripes*.
