# Supplementary material for: Purifying selection constrains the evolution of Juquitiba virus in wild Oligoryzomys nigripes communities
Source: PLoS Pathog. 2026 Jan 20;22(1):e1013839. doi: 10.1371/journal.ppat.1013839 (PMC12844527; doi:10.1371/journal.ppat.1013839)
Supplement: S8 Table — (DOCX) [file ppat.1013839.s012.docx]

**S8 Table. Parameter estimates (Estimate ± SE) for JUQV GP variants**

| **Variant** | **EC₅₀ (Dilution Factor)** | **Hill Slope (b)** |
| --- | --- | --- |
| **JUQV GP (WT)** | 3.87 ± 0.19 | 1.19 ± 0.07 |
| **JUQV GP (Q292H)** | 3.64 ± 0.18 | 1.17 ± 0.07 |
| **JUQV GP (V504I)** | 1.80 ± 0.12 | 0.90 ± 0.06 |
