# Supplementary material for: Purifying selection constrains the evolution of Juquitiba virus in wild Oligoryzomys nigripes communities
Source: PLoS Pathog. 2026 Jan 20;22(1):e1013839. doi: 10.1371/journal.ppat.1013839 (PMC12844527; doi:10.1371/journal.ppat.1013839)
Supplement: S9 Table — (DOCX) [file ppat.1013839.s013.docx]

**S9 Table. Listing of the TK numbers of 33 individual *Oligoryzomys* mice listed in Figure 1* of this paper.**

| **TK #** | **Museum** | **Catalog #** |
| --- | --- | --- |
| 66695 | MNHNP | 5743 |
| 66745 | MNHNP | 5744 |
| 132709 | MNHNP | 5745 |
| 133233 |  | REL |
| 133244 |  | REL |
| 133245 | MNHNP | 5746 |
| 140411 | MNHNP | NYC |
| 140468 |  | REL |
| 141455 |  | REL |
| 141528 |  | REL |
| 141591 |  | REL |
| 141638 | MNHNP | NYC |
| 141660 | MNHNP | 5747 |
| 141672 | MNHNP | 5748 |
| 141845 |  | REL |
| 141952 |  | REL |
| 170014 |  | REL |
| 170069 | MNHNP | NYC |
| 170224 |  | REL |
| 170226 |  | REL |
| 184699 | MNHNP | NYC |
| 184765 | MNHNP | 5749 |
| 184781 | MNHNP | 5750 |
| 184858 | MNHNP | 5751 |
| 184889 | MNHNP | 5752 |
| 184992 | MNHNP | 5753 |
| 186283 | MNHNP | NYC |
| 186318 | MNHNP | 5754 |
| 186352 | MNHNP | 5755 |
| 186353 | MNHNP | 5756 |
| 246028 | MNHNP | 5757 |
| 246028 |  | REL |
| 246099 | MNHNP | 5758 |

*All were *O. nigripes*, except for TK 66745 which was an *O. mattogrossae*. MNHNP = Museo Nacional de Historia Natural del Paraguay, where 21 voucher specimens are deposited, with the permanent museum catalog number of the voucher specimen. NYC indicates that the voucher specimen was not yet cataloged in the MNHNP as of the time of the writing of this paper. REL indicates that the mouse was released as part of a mark-release-recapture study.
